# A Cost-Effective Approach to Sequence Hundreds of Complete Mitochondrial Genomes

**DOI:** 10.1371/journal.pone.0160958

**Published:** 2016-08-09

**Authors:** Joaquin C. B. Nunez, Marjorie F. Oleksiak

**Affiliations:** University of Miami, Rosenstiel School of Marine and Atmospheric Science, Department of Marine Biology and Ecology, Miami, Florida, United States of America; International Atomic Energy Agency, AUSTRIA

## Abstract

We present a cost-effective approach to sequence whole mitochondrial genomes for hundreds of individuals. Our approach uses small reaction volumes and unmodified (non-phosphorylated) barcoded adaptors to minimize reagent costs. We demonstrate our approach by sequencing 383 *Fundulus sp*. mitochondrial genomes (192 *F*. *heteroclitus* and 191 *F*. *majalis*). Prior to sequencing, we amplified the mitochondrial genomes using 4–5 custom-made, overlapping primer pairs, and sequencing was performed on an Illumina HiSeq 2500 platform. After removing low quality and short sequences, 2.9 million and 2.8 million reads were generated for *F*. *heteroclitus* and *F*. *majalis* respectively. Individual genomes were assembled for each species by mapping barcoded reads to a reference genome. For *F*. *majalis*, the reference genome was built *de novo*. On average, individual consensus sequences had high coverage: 61-fold for *F*. *heteroclitus* and 57-fold for *F*. *majalis*. The approach discussed in this paper is optimized for sequencing mitochondrial genomes on an Illumina platform. However, with the proper modifications, this approach could be easily applied to other small genomes and sequencing platforms.

## Introduction

Mitochondrial DNA (mtDNA) sequences are used to understand metabolic disease, species diversity, demography, and evolutionary adaptation [1–8]. Due to its maternal inheritance and evolutionary dynamics, the mitochondrial genome has been a pivotal bridge between systematics and population genetics, facilitating intraspecific phylogeography studies and subsequently providing insight on the evolutionary history of both natural and human populations [9, 10]. Moreover, the mitochondrial cytochrome oxidase subunit I (COI) gene has become a widely used tool for DNA barcoding (species identification based on a specific nucleotide sequence in a species' genome, [11]). Yet, despite its nature as a robust genetic marker, most natural populations studies use only a small portion of the mitochondrial genome (*e*.*g*., the D-loop or COI gene). With today's next-generation sequencing throughput, we now can easily sequence full mitochondrial genomes. Theoretically, one can sequence 1,819 human mitochondria (16,569 bp) with 500-fold coverage using 100 bp single end reads on a single lane of a HiSeq 1500/2500 using a rapid run (<$1.00/sample; http://support.illumina.com/downloads/sequencing_coverage_calculator.html).

Not surprisingly, next-generation sequencing approaches are most cost-effective with greater sample sizes. This cost-effectiveness is readily achieved by using a multiplexed library where barcodes are used to identify each individual [12, 13]. However, kit and reagent prices often make the library preparation step much more expensive than the price of sequencing itself [14], especially for multiplexed samples. Thus, the library preparation costs become the bottleneck for how many samples one can afford to sequence.

We provide a cost-effective protocol to create multiplexed libraries and sequence whole mitochondrial genomes for many individuals. This protocol can be extended to other DNAs one might want to sequence for many individuals (*e*.*g*., chloroplasts, targeted genes, or small prokaryote genomes). We demonstrate our approach's effectiveness by generating libraries and sequencing hundreds of complete or nearly complete mitochondrial genomes (mtDNA) from killifish species *Fundulus heteroclitus* and *Fundulus majalis*. *F*. *heteroclitus* is a small, euryhaline, non-migratory [15] coastal killifish with large effective population sizes, and stable structured populations along the North American east coast. Its widespread distribution and high site fidelity have established this fish as a valuable system to investigate biological responses to a variety of environmental stressors [16] such as pollution [17–21], temperature [22–26], hypoxia [27], and salinity changes [28]. Moreover, the mitochondrial genome of this killifish displays two characteristic haplotypes corresponding to distinctly distributed clades along the North American east coast with a phylogenetic break and concurrent admixture zone found at the Hudson River [29]. *F*. *majalis* is also an euryhaline killifish inhabiting the east coast of the United States. It is a less developed system than *F*. *heteroclitus*; however, it has been used in hybrid zone studies [30].

## Methods

### Samples and mtDNA amplification

*Fundulus heteroclitus* and *Fundulus majalis* samples were collected from locations in Massachusetts using minnow traps, fin clipped and released. Fin clips were stored in Chaos, a chaotropic buffer (4.5M guanidinium thiocyanate, 2% N-laurylsarcosine, 50mM EDTA, 25mM Tris-HCL pH 7.5, 0.2% antifoam, 0.1M β-mercaptoethanol) until use. Genomic DNA was isolated from fin clips using silica column (EconoSpin(TM) All-in-1 Mini Spin Columns for DNA/RNA extraction; as described in [31]). Genomic DNA quality was assessed *via* gel electrophoresis and concentrations were quantified using Biotium AccuBlue^TM^ Broad Range dsDNA Quantitative Solution according to the manufacturer’s instructions.

**Fig 1:**
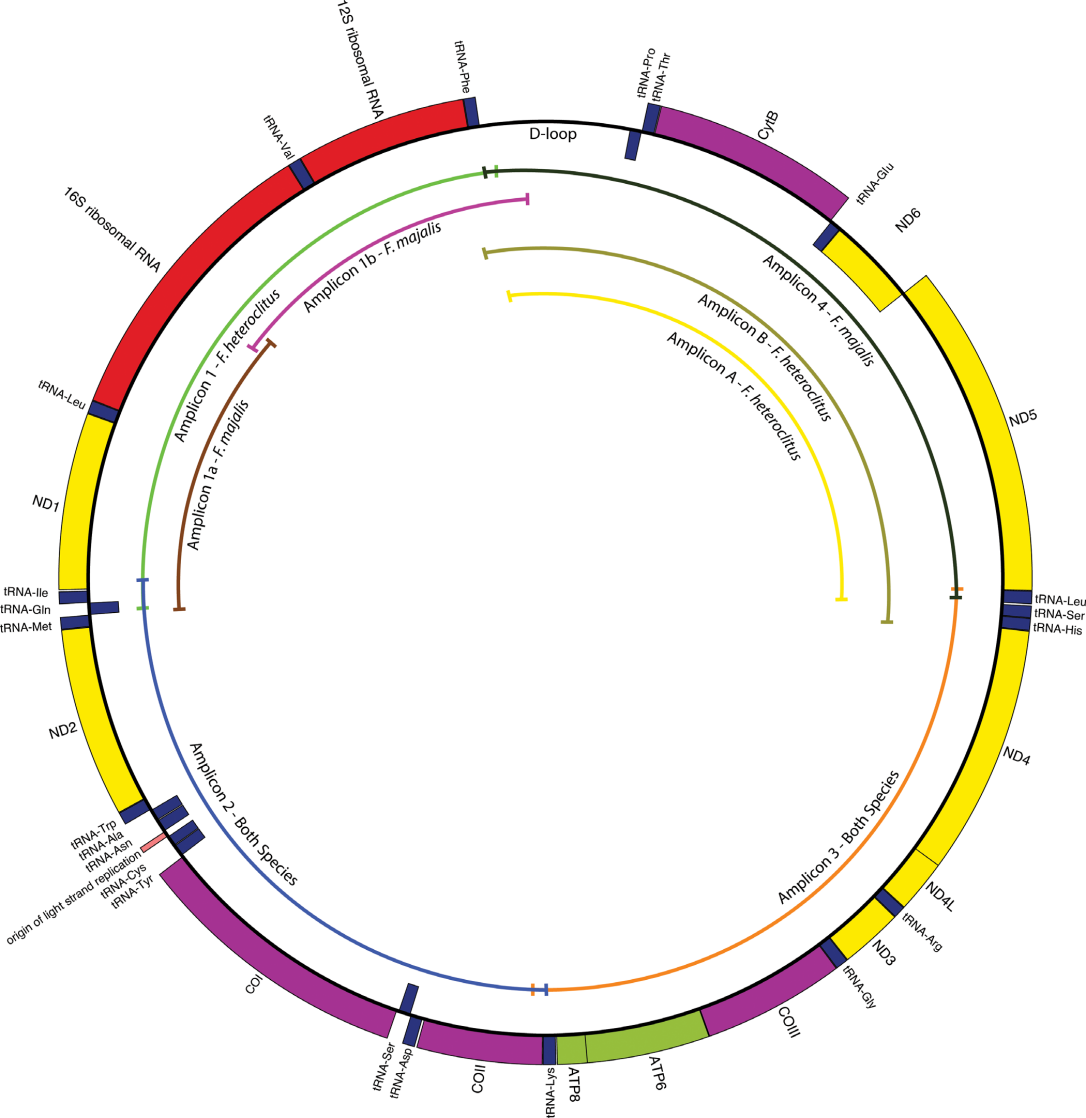
Complete *F*. *heteroclitus* mitochondrial genome. Fragments (amplicons) used to PCR amplify the mitochondrial sequence. The primers used for *F*. *majalis* were designed using *F*. *heteroclitus* as a template.

Mitochondrial genomes were amplified in four or five, overlapping, 2,000–5,000 bp PCR products using custom primers (Table 1; Fig 1; genome drawings were generated using OGDRAW by [32]) designed based on aligned *Fundulus* mitochondrial sequences available in NCBI (GeneBank numbers respectively: FJ445394.1, FJ445395.1, FJ445400.1, FJ445401.1, FJ445396.1, FJ445397.1, FJ445398.1, FJ445399.1, FJ445402.1, FJ445403.1, NC_011380.1; [33]). Amplification and sequencing of the two overlying *F*. *heteroclitus* primer sets for the fourth amplicon was an oversight. Amplification conditions were 35 cycles of denaturation at 94° C for 10 seconds, annealing at 52–57° C (Table 1) for 30 seconds, and extension at 72° C for 5 minutes using 2.5 U Taq (Promega GoTaq®) polymerase, 200 uM dNTPs, 0.2 uM each primer, and ~50–100 ng genomic DNA in 50 mM Tris HCl pH 9.2, 16 mM NH_4_2SO_4_, 2.25 mM MgCl_2_, 0.1% tween, and 2% DMSO.

**Table 1:** Primers utilized to amplify the complete mitochondrial genome.

Amplicon	Species	Position	Sequence (5' to 3')	Amplicon Size	Annealing T°C
1	*heteroclitus*	16095F	GGCATTTGGTTCCTATTTCAGG	4449	55
1	*heteroclitus*	3981R	GCTGACTTTACTAGAAAATGGTGTAG		
2	*heteroclitus/majalis*	3929F	TCAAAACTCTTGGTGCTTCC	3952	55
2	*heteroclitus/majalis*	7880R	CTTCTTAACGAGGCGTCTTC		
3	*heteroclitus/majalis*	7850F	TAGAACACTTCGAAAACTGATC	4122	52
3	*heteroclitus/majalis*	12060R	TGCTACTTGGAGTTGCACC		
4a	*heteroclitus*	11471F	TCGAAAGCAACGATGACTGC	4423	55
4a	*heteroclitus*	16250R	TGTATGCACTGTGAAATGTCAAC		
4b	*heteroclitus*	11800F	TCTTATTCACCGAGAGAGGC	4344	55
4b	*heteroclitus*	16150R	ATTCCACCATTAACTTATGC		
1a	*majalis*	2028F	CCTGTTTACCAAAAACATCGCC	2012	55
1a	*majalis*	4000R	CCAACATGTTCGGGGTATG		
1b	*majalis*	16095F	GGCATTTGGTTCCTATTTCAGG	2596	55
1b	*majalis*	2098R	ATTGCGCTACCTTTGCACG		
4	*majalis*	11915F	TCATCCGTTGGTCTTAGGAACC	4235	57
4	*majalis*	16150R	ATTCCACCATTAACTTATGC		

Primers utilized to amplify mtDNAs of *F*. *heteroclitus* and *F*. *majalis*

For each individual, equal volumes of the PCR products were pooled prior to purification. Ideally, to better control sequencing coverage across the mitochondrial genome, one would purify and quantify each PCR product and then combine equal molar amounts of the different PCR products. However, because most PCR fragments were similar sizes and the products showed similar intensities when resolved on agarose gels, we opted to save time and reagents and pool samples based on volume rather than quantified molar amounts.

After pooling PCR products, amplified PCR products were purified using an equal volume of carboxyl coated magnetic beads (Fisher Scientific) in a PEG/salt solution (0.5 g beads in 100 ml of 20% PEG 8000, 2.5 M NaCl; [34]) and quantified as described above using Biotium AccuBlue^TM^ Broad Range dsDNA Quantitative Solution. Then, 100 ng of purified DNA from each sample was dried down in a 96-well format to use for library preparation. Drying a constant DNA amount and reconstituting it in a set volume is easier than diluting different concentrations to the set concentration needed for processing the library in a 96-well format.

### Illumina HiSeq Library Construction

The following is a simplified protocol for library preparation; a detailed protocol is available as S1 File. In brief, dried DNA samples were reconstituted overnight using 2 μl of H_2_O. MtDNA amplification products were fragmented using 0.5 μl of NEBNext^®^ dsDNA Fragmentase^®^ (New England Biolabs, Ipswitch, MA) in 5 μl volumes for 30 minutes at 37° C followed by a 4° C soak. Samples were then diluted with 45 μl of 100 mM EDTA and 10 mM Tris pH 7.8. Fragments smaller than 300 bp were selected using two, sequential DNA bead purifications [35]. In this process, the PEG and salt concentrations determine the fragment sizes that bind to the beads. First, to bind fragments bigger than 300 bp to the beads, 0.55 volumes (27.5 μl) of magnetic beads in 20% PEG 8000 and 2.5 M NaCl were added to each sample. After binding for 10 minutes, beads were magnetized, and the supernatants containing fragments less than 300 bp were removed to a new plate. To purify fragments between 100 bp and 300 bp, the same volume of beads (27.5 μl) was added to each supernatant (thus increasing NaCl and PEG concentrations) and allowed to bind DNA for 10 minutes. Beads were subsequently magnetized, the supernatants were removed, and beads were washed two times with 70% ethanol. After the final wash removal, beads were air dried 2–5 minutes. Notice, DNA is not yet eluted from the beads. This fragmentation and size selection yields ~10% of the starting material or ~10 ng based on spectrophotometry.

Individuals were prepared for barcoding by adding 0.4 pmol of adaptors to each sample in a 4 μl volume (adaptors were added directly to the DNA bound beads). For mitochondrial DNA sequencing, we re-purposed adapters used for genotyping by sequencing (GBS; [13]). These adapters consist of two adapters. The first adapter has a 4 to 8 base pair barcode on the end of its top strand and a 2 base pair overhang on the end of the bottom strand that is complementary to the overhang left by digestion with a restriction enzyme. Barcodes are listed in the supplementary bioinformatics pipeline, and all pair-wise barcode combinations differed by at least three mutational steps to minimize sample misidentification due to barcode synthesis or sequencing errors [13]. The second adapter is a common adapter that just has the 2 base pair overhang. These adapters allow for either single-end or paired-end sequencing on the Illumina Next Generation Sequencing platforms [13]. However, because they have a 2 bp overhang, they need to be blunted before they can be ligated to the mtDNA, which is randomly fragmented. We end repaired (blunted and kinased) both the fragmented mtDNA and adaptors in the same reaction.

To blunt and kinase both the barcodes and DNA fragments, the fragmented DNA, bead and adaptor mix was combined with 6 μl of an end-repair master mix and incubated at 20° C for 30 minutes. The end-repair master mix contained 1.67x ligation buffer (New England Biolabs), 0.67 mM dNTPs, 5 U of T4 polynucleotide kinase (New England Biolabs), and 1.5 U T4 DNA polymerase (New England Biolabs). After incubation, the polymerase was heat killed at 75° C for 20 minutes. Combining the adaptors with the DNA samples prior to end-repair allowed us to use barcodes with 2 bp overhangs also used for GBS, enhancing their utility and reducing costs. However, one could also synthesize adaptors without any overhangs if adaptors were only needed for mitochondrial sequencing.

To ligate the adaptors to the DNA fragments, 3 μl of a ligation master mix was added to each sample. The ligation master mix contained 1x ligation buffer and 100 U T4 DNA ligase (New England Biolabs). Ligation was performed at 16° C for 12–14 hours. Post-ligation, volumes were increased to 40 μl with the addition of 27 μl of TE. To purify the ligated DNA, 40 μl of PEG/salt (20% PEG 8000 and 2.5 M NaCl) was mixed with each sample. DNA was allowed to bind to the beads for 10 minutes, beads were magnetized, supernatants were removed, and remaining beads were washed 2 times with 70% ethanol. After final ethanol wash removal, beads were allowed to dry 2–5 minutes. Finally, DNA was eluted from beads with 30 μl of 0.1x TE (10 mM Tris pH .8 and 0.1 mM EDTA). An equal volume from each sample was pooled, and the final pool was concentrated ten-fold by repeating bead purification and eluting in 1/10^th^ the starting volume. Alternatively, unpurified ligation products can first be pooled and then simultaneously purified and concentrated.

Pooled samples (1 μl) were PCR amplified (using barcode adapters as priming sites) in a 50 μl final volume of 1x Taq Master Mix (New England Biolabs) for 22 cycles with 20 pmol each of 5'AATGATACGGCGACCACCGAGATCTACACTCTTTCCCTACACGACGCTCTTCCGATCT and 5'CAAGCAGAAGACGGCATACGAGATCGGTCTCGGCATTCCTGCTGAACCGCTCTTCCGATCT. PCR conditions were 98° C for 5 minutes followed by 22 cycles of 98° C for 30 seconds, 65° C for 30 seconds, and 72° C for 30 seconds and a final 5 minute soak at 72° C. PCR products were purified using 0.7x volumes of magnetic beads to isolate fragments > 150 bp and then sized on an Agilent Technologies 2100 Bioanalyzer before being sequenced. The mtDNA library was sequenced on an Illumina HiSeq 2500 with a 100 bp single end read (Elim Biopharmaceuticals, Inc.) as part of a larger sequencing effort, the *F*. *heteroclitus* samples (named CG1 through CG192) and the *F*. *majalis* samples (named CG193 through CG384) made up ~57% of the sequenced samples. Cluster density for this library was ~197M reads before passing filter, but only 54% of the data passed filter, leaving only ~107M reads. However, because the sequencing facility could not provide us with an explanation for why only 54% of the data passed filter, we analyzed all data, including pre-filter data. Reads corresponding to this sequencing run are available in the NCBI SRA archive as PRJNA291658 (*Fundulus majalis*) and PRJNA291654 (*Fundulus heteroclitus*). We have also included the individual SRRs and other metadata for each sample as S2 File.

Table 2 summarizes library preparation costs for whole mitochondrial sequencing using the Illumina sequencing platform. Costs are based on list prices and do not include labor costs or sample preparation costs, which will vary depending on how mtDNA is isolated (for us, mitochondrial DNA amplification costs were approximately $0.94/sample). Our cost to prepare 96 mtDNAs for Illumina sequencing was $158.48 or $1.65/sample (Table 2).

**Table 2:** Library Preparation Costs.

**Reagent**	**Amount**	**Cost per mitochondria**	**Cost per 96 mitochondria**
Fragmentase	0.5 μl	0.392	$37.63
dNTPs	4 μl of 1 mM mix	0.025	$2.40
T4 polynucleotide kinase	0.25 μl	0.224	$21.50
T4 DNA polymerase	0.25 μl	0.26	$24.96
T4 DNA ligase	0.25 μl	0.256	$24.58
Magnetic carboxy beads	50 μl	0.045	$4.32
Adaptors	~85 bp	0.000272	$0.03
**Total Reagents**		**$1.202272**	**$115.42**
**Plastics**			
Racks of tips	8		$34.63
96-well plates	2		$8.43
2x Taq Master Mix		0.28	$0.28
**TOTAL COST**			**$158.48**

Approximate library preparation costs for whole mitochondrial sequencing using the Illumina sequencing platform.

### Sequence Alignment and Genome Investigations

The detailed bioinformatics pipeline is included as S3 File. Illumina adaptor sequences, short sequences (<50 bp), and sequences with low Phred scores (<20) were trimmed using trim_galore v0.3.7 (http://www.bioinformatics.babraham.ac.uk/projects/trim_galore/) and cut adapt v1.9 [36]. Individuals were sorted into separate files based on barcodes; the barcodes sites were trimmed using a custom Unix script (see S3 File). The quality of resulting reads was assessed using FastQC v0.11.4 (http://www.bioinformatics.babraham.ac.uk/projects/fastqc/). High quality reads from each sample were aligned individually to their respective reference genome (see below) using Bowtie2 v2.2.3 [37]. Consensus sequences were generated using SAMtools v1.2, BCFtools v1.2, and VCFutils [38, 39]. *F*. *heteroclitus* sequences were aligned to a reference *F*. *heteroclitus* mitochondrion genome (*F*. *heteroclitus* isolate 02 pop-variant ME mitochondrion, complete genome; GenBank: FJ445403.1). In contrast, the *F*. *majalis* sequences were aligned to a reference genome constructed *de novo* using MITObim [40], and reads from a single *F*. *majalis* individual. To further test the effectiveness of this approach, we also assembled a *de novo* mtDNA from reads from a single *F*. *heteroclitus* individual.

The *F*. *majalis* sample for *de novo* assembly was chosen based on read quality (measured in phred score) and number of reads available (see results). We constructed two independent *F*. *majalis de novo* genomes using 2 different sequence baits. The first bait was the 989 bp *F*. *majalis* CytB gene sequence available in NCBI (GenBank: GQ119735.1). The second bait was the complete *F*. *heteroclitus* mitochondrial genome, also available in NCBI (GenBank: FJ445403.1). These bait/iteration processes ran with 900 and 115 proposed iterations (k = 31 for both runs) and converged at 105 and 27 iterations respectively. The *de novo F*. *heteroclitus* genome was reconstructed independently from 1 sequence bait: a 989 bp portion of the *F*. *heteroclitus* CytB gene (the same region used in the *F*. *majalis* run) sequence available on NCBI (GenBank: FJ445403.1 from 14414 to 15402). Baiting with CytB ran with 900 proposed iterations and k = 31. The algorithm converged at 194 iterations. The resulting *de novo* genomes were evaluated using the NCBI Basic Local Alignment Search Tool (BLASTn). BLASTn searches were conducted against the *Fundulidae* database (taxid: 28756), and optimized for highly similar sequences (megaBLAST). All other parameters were used as default. Individual alignments were visualized using Tablet [41]. Quality control for the alignments were performed in Qualimap v2.0.1 [42]. *De novo* contigs using unmapped reads were built with SPAdes in unipro Ugene [43, 44]. The number of polymorphic sites was calculated in Arlequin v.3.5.1.2 [45].

Phylogenetic inferences were estimated in RAxML [46] using the GTR-Γ model of molecular evolution, 1000 bootstraps (-N 1000), and a consistent pseudo-random seed of 12345 for options -p and -m. Tree visualization and annotation was done in the R package ggtree (http://bioconductor.org/packages/release/bioc/html/ggtree.html) and FigTree v.1.4.2. (http://tree.bio.ed.ac.uk/software/figtree/). An unrooted phylogenetic tree was constructed using only the samples sequenced in this experiment; for this tree, we filtered our data sequences with high levels of missing data (>80%), thus keeping 302 sequences from both species. Furthermore, we only kept variant loci present in 99% of remaining individuals for a total of 5,088 loci distributed along the mtDNA. Filtering was done using TASSEL 5 v5.2.27 [47]. We also constructed two gene trees with our data and publically available data. A gene specific tree for the Cytochrome Oxidase I gene (COI) was constructed using the region corresponding to COI in all samples, the *Fundulus majalis’* COI barcode sequence (TDWGP005-11 from BOLD systems v3 [48]), and the following COI portion NCBI published mtDNAs: *Fundulus heteroclitus* (GenBank: FJ445403.1), *Fundulus notatus* (GenBank: NC_028293.1), *Fundulus diaphanus* (GenBank: NC_012361.1), *Fundulus grandis* (GenBank: FJ445397.1), *Fundulus olivaceus* (GenBank: NC_011380.1). Samples CG161, CG137, CG114, CG44, CG32, CG84, CG205, CG233, CG319, CG340, CG343, CG347, CG359, and CG372 had high levels of missing data (>99%) and were excluded from the analysis. A gene specific tree for the cytochrome B gene was constructed using the region corresponding to CytB in all samples, the F. majalis CytB (GenBank: GQ119735.1). The CytB sequences from all other *Fundulus* species were extracted from the same GenBank records as the COI reconstruction. Samples CG343, CG233, CG347, and CG352 had high levels of missing data (>99%) and were excluded from the analysis. In both cases, gap regions generated at the edge of the multiple sequence alignment were trimmed. This light filtering of missing data has been shown to slightly improve phylogenetic signal [49].

### Ethics Statement

Fish were collected within publically available lands and no permission was required for access. *Fundulus heteroclitus* and *Fundulus majalis* are not endangered or protected species, and do not require collecting permits for non-commercial purposes. All fish were captured in minnow traps with little stress and released back to the site of capture in less than an hour. For all individuals, only a small sample tissue (fin clip < 4mm x 4mm) was collected. The University of Miami IACUC approved this study.

## Results

We sequenced 383 mitochondrial genomes using Illumina HiSeq 2500: 192 *Fundulus heteroclitus* samples and 191 *Fundulus majalis* samples. The raw output resulted in 2.99 million *F*. *heteroclitus* reads distributed across 192 individual files and 2.88 million *F*. *majalis* reads distributed across 191 individual files after removal of adaptors (including adaptors inappropriately ligated to inserts, see S3 File), barcodes, low quality sequences (phred score < 20), and sequences shorter than 50 bp.

### Mapping *F*. *heteroclitus* reads to a reference mtDNA genome

**Fig 2:**
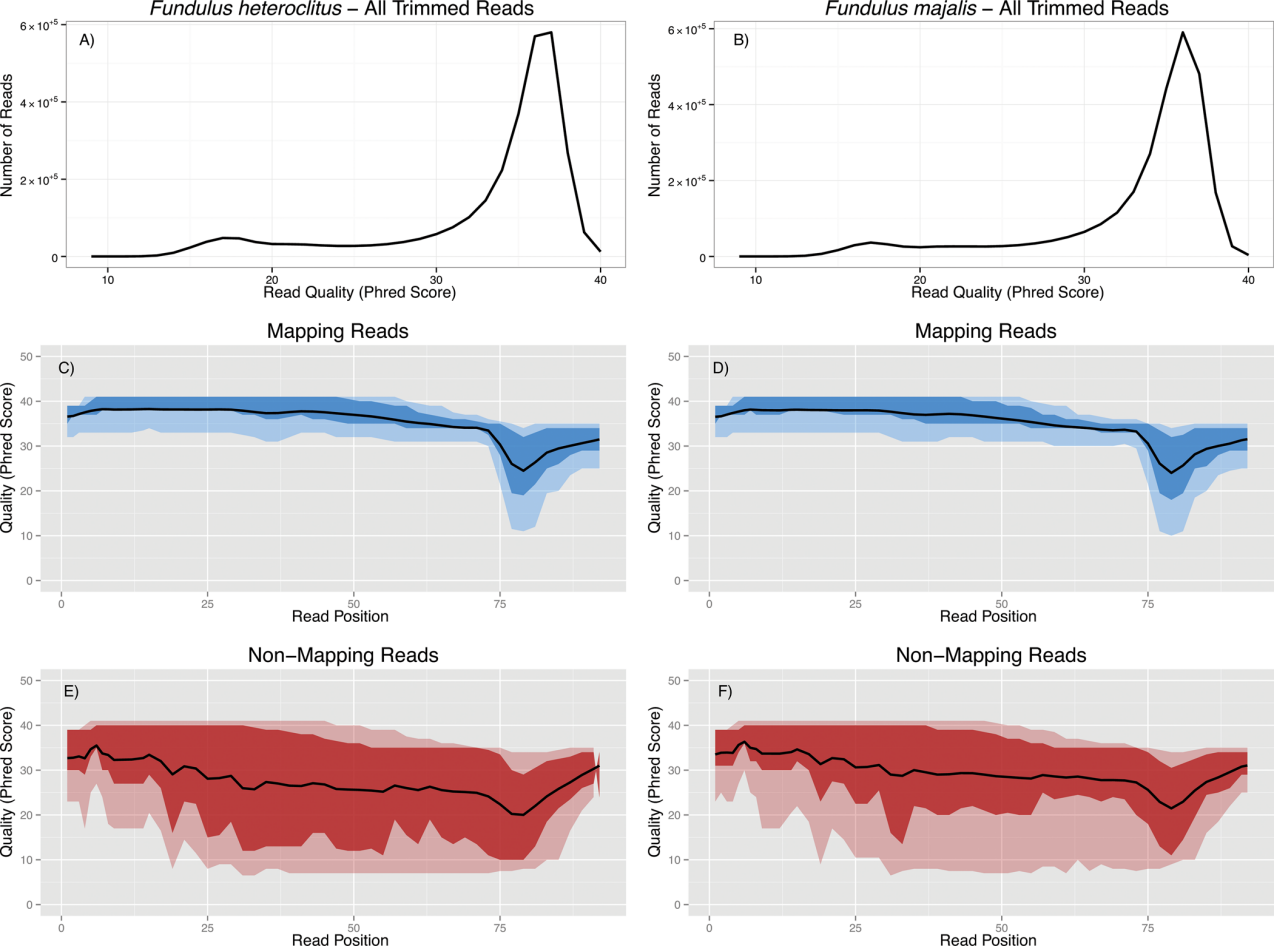
Quality control measurements of raw reads for *F*. *heteroclitus* (A, C, E) and *F*. *majalis* (B, D, F). A-B) Figure shows number of all available reads after trimming *vs*. read size. Nucleotide window analyses of *F*. *heteroclitus* aligned reads (C) and unaligned reads (E). Nucleotide window analyses of *F*. *majalis* aligned reads (D) and unaligned reads (F). The outer ribbon of low opacity represents the 10^th^ and 90^th^ quality percentiles. The inner opaque ribbon represents the upper and lower quality quartiles. Black lines represent averages.

The 2,991,394 *F*. *heteroclitus* sequences remaining after barcode trimming were 50 to 92 bp in length (average = 84.8 bp; GC% = 46). The average phred score among all reads was 33.1 (s.d. = 5.90, Min = 9, Max = 40; Fig 2A). For the 192 individual *F*. *heteroclitus* sequences, on average, 75.26% of the reads mapped to the *F*. *heteroclitus* mitochondrial genome (range: 10.23% to 94.35%) using Bowtie2. The average mapped read length was 86.1 bp. The mapped read phred score was 35.10 (s.d. = 2.87, Min = 13, Max = 40; Fig 2C). Reads that did not map to the reference had an average length of 81.35 bp (s.d. = 9.20 bp) and an average phred score of 27.19 (s.d. = 8.30, Fig 2E). A nucleotide window analysis shows that all reads have low phred scores towards the middle of the read; however, the 3’ and 5’ ends show high phred scores (Fig 2C and 2E). These flanking, high phred score regions resulted in otherwise bad quality reads not being filtered in the quality trim step (see discussion).

**Fig 3:**
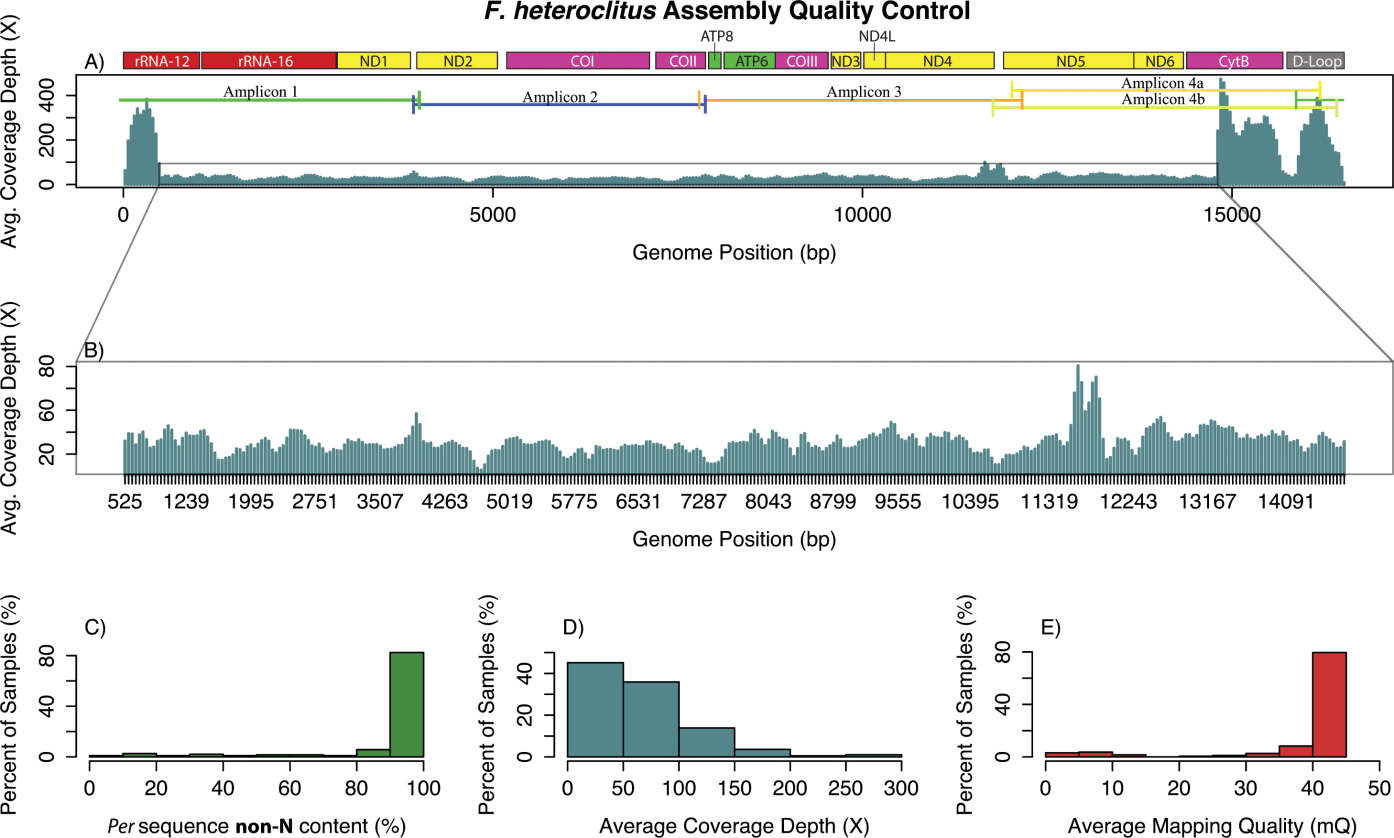
Quality control information for *Fundulus heteroclitus* samples. A) Average coverage depth along the mtDNA genome. Amplification products (amplicons) are shown across the top. B) Amplified view of the average, *F*. *heteroclitus* mitochondrial genome, coverage depth. C) Percent of samples (out of 192) showing non-N nucleotides. D) Percent of samples showing different coverage depths. E) Percent of samples showing different mapping quality levels.

*F*. *heteroclitus* assembled mtDNAs showed an average per sequence non-N content of 91.26% (*i*.*e*., on average, sequences had 8.7% or 1,445 Ns) with most individuals (107 out of 192 or 56%) having 100% non-N content (Fig 3C). The mean coverage depth for all individuals was 61.7-fold (s.d. = 89.7) (Fig 3D). However, this mean is biased due to over-representation of regions at the ends of the reference mtDNA genome. Excluding the over-represented regions, the majority of the genomic regions for individual alignments showed an average coverage close to ~30-fold (Fig 3A and 3B). The mapping quality (mQ) values ranged from 1.47 to 41.51 with an average of 37.8; however, 75% of the samples showed an mQ > 41 (Fig 3E). We investigated the regions with high coverage depth *versus* those with close-to-average coverage depth by assaying the number of polymorphic sites for each region. Regions with high coverage spanned a 2,179 bp long region including portions of the Cytochrome B gene, the D-loop, the 12S ribosomal gene, and portions of the 16S ribosomal gene with a short pronounced trough close to the beginning of the D-loop (Fig 3A). This high coverage region is part of amplicons 1, 4a and 4b. This region had 6.7% polymorphic sites (calculated as number of polymorphisms/length of high coverage region). Regions with close-to-average coverage depth spanned a 14,348 bp long region including most mitochondrial genes. This region showed 7.7% polymorphic sites. Additionally, the overrepresented regions did not show any particular correlation with GC content.

### De novo assembly of *F*. *majalis* reference mtDNA

The 2,882,285 *F*. *majalis* reads remaining after barcode trimming were 50 to 92 bp (mean = 84.7 bp; GC% = 45). The average phred score among all reads was 33.18 (s.d. = 5.34, min. = 9, max. = 40; Fig 2B). For *Fundulus majalis*, the reference mitochondrial genome was assembled *de novo* using MITObim [40]. The sample with barcode CG224 was selected as read pool to MITObim due to its high read quality and number of reads. The fastq file contained 46,152 reads sizing from 50 to 91 bp (GC% = 44). The average phred score among all reads was 34.39 (s.d. = 3.68). FastQC flagged no sequences as ‘bad quality’.

*De novo* assembly of *F*. *majalus* reads using the *F*. *majalus* CytB gene as bait resulted in a single contig 9,763 bp long; 33,839 (60%) reads were used in the assembly, and the coverage was 306.79-fold. GC content was 42.01%. *De novo* assembly with the full *F*. *heteroclitus* mtDNA as bait also resulted in a single contig 16,812 bp long; 37,678 (67%) reads were used in the assembly, and the average coverage was 202.79-fold. GC content was 42.15%. The latter assembly, however, contained a semi-contiguous region of 2,842 undefined nucleotides (‘Ns’). With the exception of three 150–400 bp interspersed regions with non-N nucleotide sequences, the missing region spanned 3,900 bp in the assembly from positions 8,000 to 11,900 bp.

The genome generated using the CytB bait was searched against the NCBI database using BLASTn. Search results returned the 12 highest hits to mitochondrial genomes of *Fundulus* taxa. The six highest hits map to *Fundulus heteroclitus* mtDNA, followed by *Fundulus diaphanus*, *Fundulus grandis*, *Fundulus notatus*, and *Fundulus olivaceous* mtDNAs. All these hits had corresponding 0 E values, 86% query cover and identity scores ranging from 85% to 87%. The top hit, against the mtDNA of *Fundulus heteroclitus* isolate 02 pop-variant GAS (GenBank: FJ445401.1), revealed that the *de novo* mtDNA spanned a region from the tRNA-leu gene (*F*. *heteroclitus* reference position 11,885) to the tRNA-Ile gene (*F*. *heteroclitus* reference position 3,824) going around the circular genome. The entire region spanning positions 3,825 to 11,884 (in the *F*. *heteroclitus* reference genome) was not reconstructed *de novo* using the CytB bait.

The genome generated using the *F*. *heterolitus* complete mtDNA bait was searched against the NCBI database using BLASTn and returned similar results as the previous *F*. *majalis de novo* genome. The 12 highest hits mapped to *Fundulus heteroclitus*, *Fundulus diaphanus*, *Fundulus grandis*, *Fundulus notatus*, and *Fundulus olivaceous* mitochondrial genomes. All these hits had corresponding 0 E values, query coverage ranging from 73% to 77%, and identity scores ranging from 85% to 88%. The top hit was the mtDNA of *Fundulus heteroclitus* isolate 02 pop-variant MDPP (GenBank: FJ445399.1).

Mapping to functional regions showed that the non-N portions of the assembly correspond to regions from the rRNA genes to the tRNA-Lys gene (*F*. *heteroclitus* reference positions 0 to 7,956), and from the tRNA-Ser to the D-loop (*F*. *heteroclitus* reference positions 11,859 to 16,526). Due to the high number of Ns in the sequence, the region spanning from 7,957 to 11,858 (corresponding to the region with Ns in the assembly) did not produce any hits. However, the small non-N regions interspersed throughout the region produced hits to loci in within the 7,957 to 11,858 region of the reference *Fundulus heteroclitus* 02 pop-variant MDPP genome. With the exception of the missing 7,957 to 11,858 bp region in the CytB generated genome, both *de novo* generated *F*. *majalis* genomes were identical. The assembly generated with the full *F*. *heteroclitus* mtDNA as a bait was used as the *F*. *majalis* reference genome.

To further test the effectiveness of the MITObim approach, we also reconstructed the *F*. *heteroclitus* mtDNA *de novo*. For this assembly, the sample with barcode CG82 was selected. The fastq file contained 30,291 reads sizing from 50 to 89 bp (GC% = 43). The average phred score among all reads was 33.04 (s.d. = 6.01). FastQC flagged no sequences as ‘bad quality’. The *F*. *heteroclitus de novo* genome was reconstructed from 1 sequence bait: a portion of the CytB gene sequence of *F*. *heteroclitus* available on NCBI (GenBank: FJ445403.1 from 14414 to 15402). *De novo* assembly resulted in one single contig 16,607 bp long; 23,971 (50.47%) reads were used in the assembly, and the coverage was 131.99-fold. The GC content was 39.81%. The assembly had no undefined (N) sites.

Search against the NCBI database resulted in the 12 highest hits mapping to *Fundulus heteroclitus*, *Fundulus diaphanus*, *Fundulus grandis*, *Fundulus notatus*, and *Fundulus olivaceous* mitochondrial genomes. All these hits had corresponding 0 E values, query cover ranging from 99% to 100%, and identity scores ranging from 84% to 99%. The top hit was *F*. *heteroclitus* mtDNA isolate 02 pop-variant ME mitochondrion (GenBank: FJ445403.1) with a coverage query = 100% and identity score = 99%. Relative to the reference genome, the assembled *de novo* genome’s position 0 is located at the ND5 gene (position 12,601 in NCBI reference genome). The *de novo* genome shows an overlap of 79 bp from 14,757 to 14,834 consisting of a short duplicated CytB segment. After removing this duplication, the corrected *de novo* assembly size is 16,528 bp, which corresponds closely to the *F*. *heteroclitus* genome sizes contained in NCBI (16,527 bp).

### Mapping *F*. *majalis* reads to the reference mtDNA genome

We prepared a library containing 192 *F*. *majalis* samples, however one sample, CG233, yielded no reads. For the remaining 191 individual *F*. *majalis* sequences, on average, 73.02% of the reads mapped to the *F*. *majalis* mtDNA using Bowtie2 (range: 25.80% to 95.45%). The average mapped read length was 86.34 bp (s.d. = 20.01), and the average mapped read phred score was 34.65 (s.d. = 2.19; Fig 2D). Reads that did not map to the reference had an average length of 83.01 bp (s.d. = 12.21 bp) and an average phred score of 29.17 (s.d. = 7.78; Fig 2F). Similar to *F*. *heteroclitus*, a nucleotide window analysis also showed a low quality region occurring at the center of most reads.

**Fig 4:**
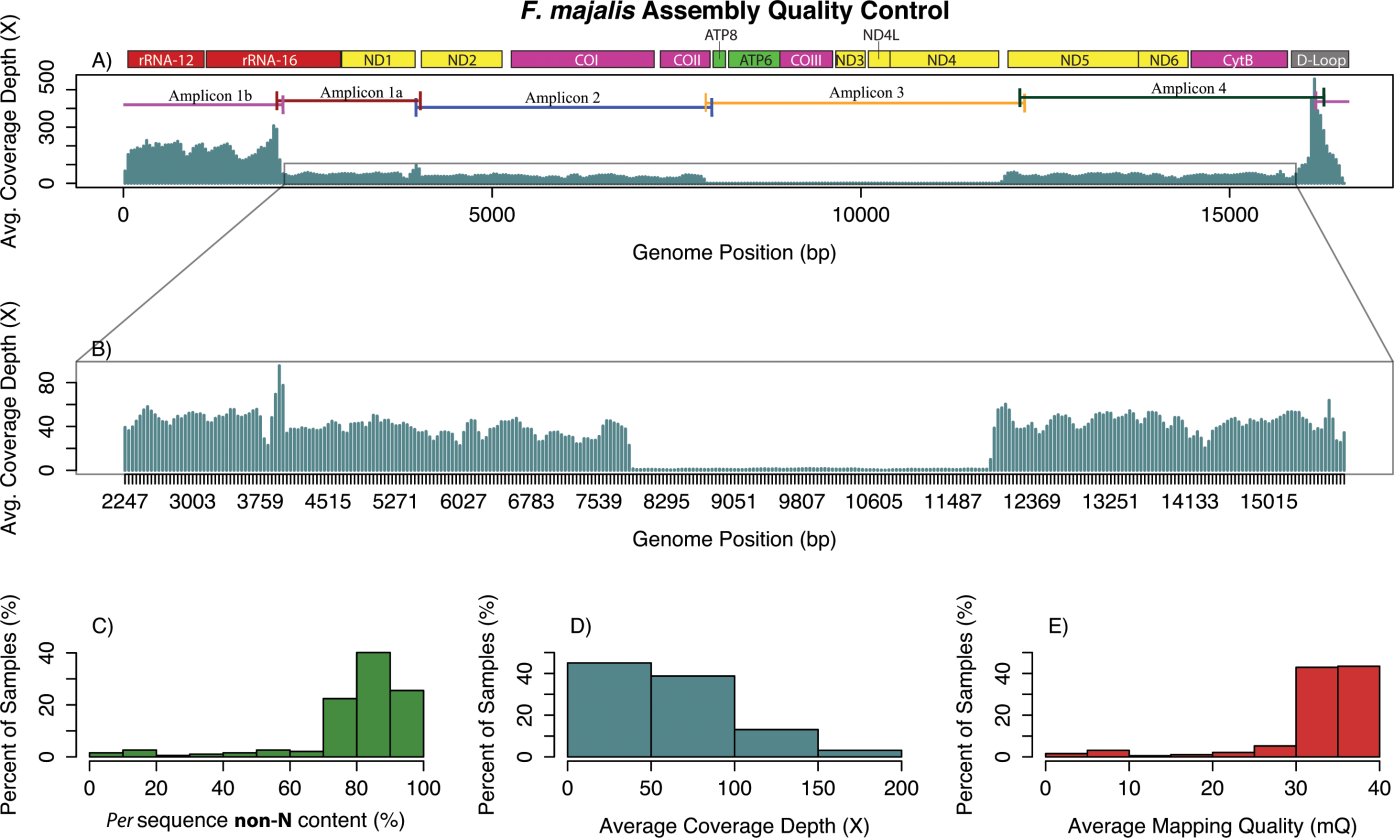
Quality control information for *Fundulus majalis* samples. A) Average coverage depth along the mtDNA genome. Amplification products (amplicons) are shown across the top. B) Amplified view of the average, *F*. *majalis* mitochondrial genome, coverage depth. C) Percent of samples (out of 191) showing non-N nucleotides. D) Percent of samples showing different coverage depths. E) Percent of samples showing different mapping quality levels.

*F*. *majalis* assembled mtDNAs showed an average per sequence non-N of 79% (*i*.*e*., on average, sequences had 21% or 3,531 Ns) with 48 of the 191 individuals showing more than 90% non-N sequences (Fig 4C). The average coverage for the samples was 57.5-fold (s.d. = 73.9) (Fig 4D). However, average coverage was affected by over-representation of the regions at the ends of the genome (as seen in *F*. *heteroclitus*) and by a low coverage window seen between 7,957 bp and 11,858 bp (Fig 4A and 4B). Excluding the over-represented and low coverage region, the average coverage for the remaining nucleotide windows was close to 43-fold. The average mapping quality for these samples was 32.7, and 87% of the samples had mapping qualities ranging from 30 to 39 (Fig 4E). For *F*. *majalis* samples, a 2,492 bp long region including the D-loop, the 12S ribosomal gene, and portions of the 16S ribosomal gene had high coverage (Fig 4A). This high coverage region, unlike that of *F*. *heteroclitus*, covers most of a single amplicon. This region showed 5.4% polymorphic sites. The remaining genome portions showed close-to-average coverage depth, spanned a 13,942 bp long region, and showed 7.2% polymorphic sites. Just as in *F*. *heteroclitus*, no correlation with GC content was observed.

### Additional investigations on non-mapping reads

For both *F*. *heteroclitus* and *F*. *majalis*, a relatively large percentage (~25%) of long (80–90 bp), low to acceptable quality reads remained unmapped after alignment. To determine whether or not our primers amplified products other than mitochondrial regions, we aligned all available reads to the complete *F*. *heteroclitus* genome *(http://www.ncbi.nlm.nih.gov/genome/?term=txid8078[Organism:exp])*. Mapping showed that reads mapped only to the scaffold corresponding to the mitochondrial genome in the complete *F*. *heteroclitus* genome (mean coverage ~15,000-fold).

To further investigate the unmapped reads, we pooled unmapped *F*. *heteroclitus* read files and built *de novo* contigs from these. *De novo* assembly from these reads resulted in 443 contigs. Twenty-three (5.2%) of these contigs were longer than 1,000 bp, and nine (2.0%) had BLAST hits with 0 E values and 80%-99% identity values to fragments of mitochondrial genomes from *Fundulus* taxa. However, aligning reads to any of these contigs resulted in very low alignment rates, suggesting that the reads and contigs had sequencing errors, which were preventing alignments.

Unmapped *F*. *majalis* read filtering resulted in 1.7 million reads from the original 3.8 million reads. Remapping these unmapped reads to the mtDNA reference genome resulted in 0 reads aligning to the reference. *De novo* assembly from these reads resulted in 692 contigs. Twenty-nine (4.2%) of the contigs were longer than 1,000 bp, and ten (1.44%) had BLAST hits with ~0 E values and 80%-99% identity values to a fragment of a mitochondrial genome from *Fundulus* taxa. One of these contigs with good BLAST hits mapped to the nucleotide window located between 8,000 and 11,000 bps in a *F*. *heteroclitus* mitochondrial genome (as mapped in NCBI in GenBank: FJ445403.1). This nucleotide window corresponded to the area with the lowest coverage in the *F*. *majalis* alignment. However, aligning reads to any of the aforementioned contigs, including the one resembling the missing genome window, resulted in very low alignment rates, again suggesting that the reads and contigs had sequencing errors, which were preventing alignments.

### Sequence Investigations

**Fig 5:**
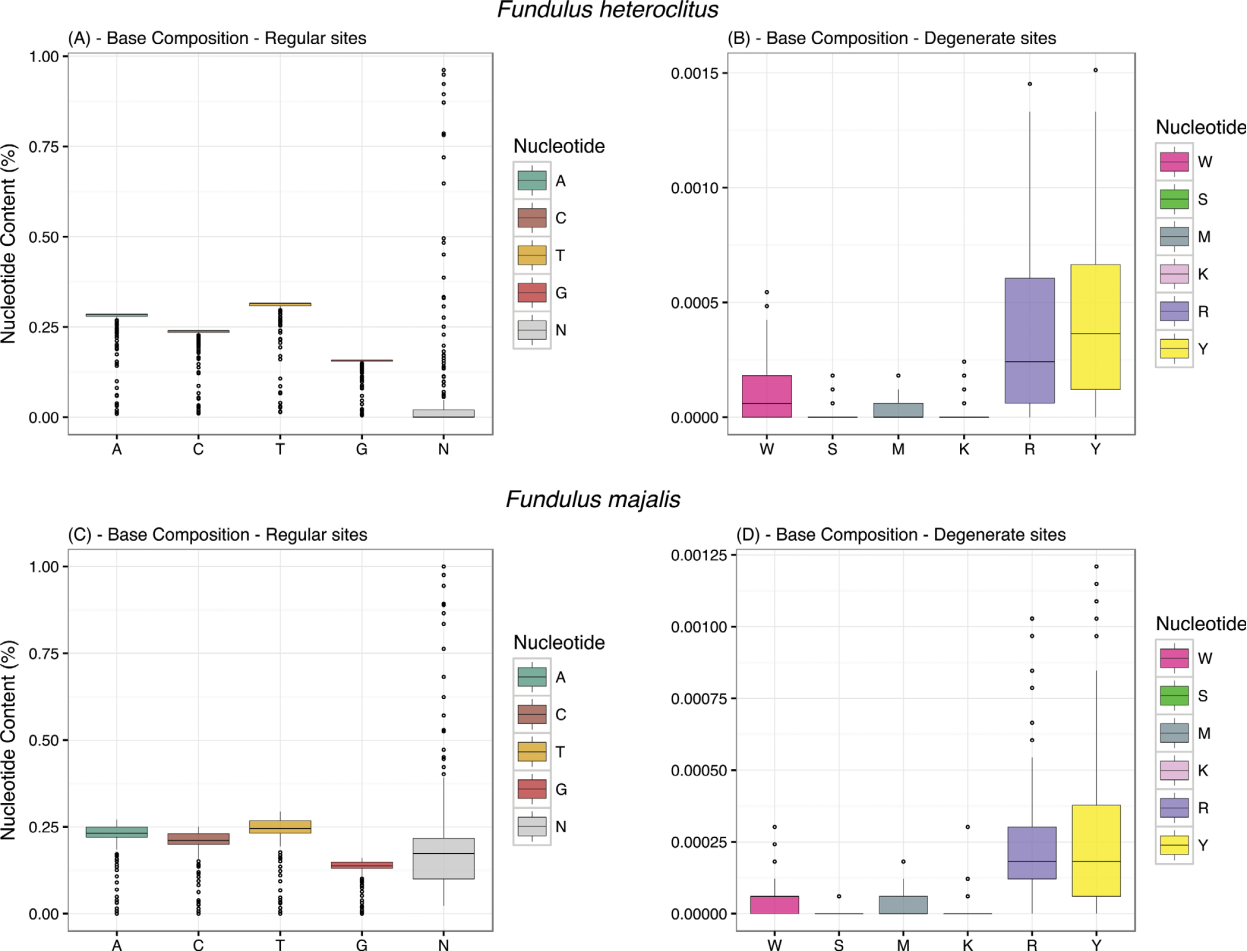
Base composition across all sequences. Regular calls (A,C,T,G, or N) in A) *F*. *heteroclitus*, and C) *F*. *majalis*. Degenerate calls (W,S,M,K,Y) in B) *F*. *heteroclitus*, and D) *F*. *majalis*.

**Fig 6:**
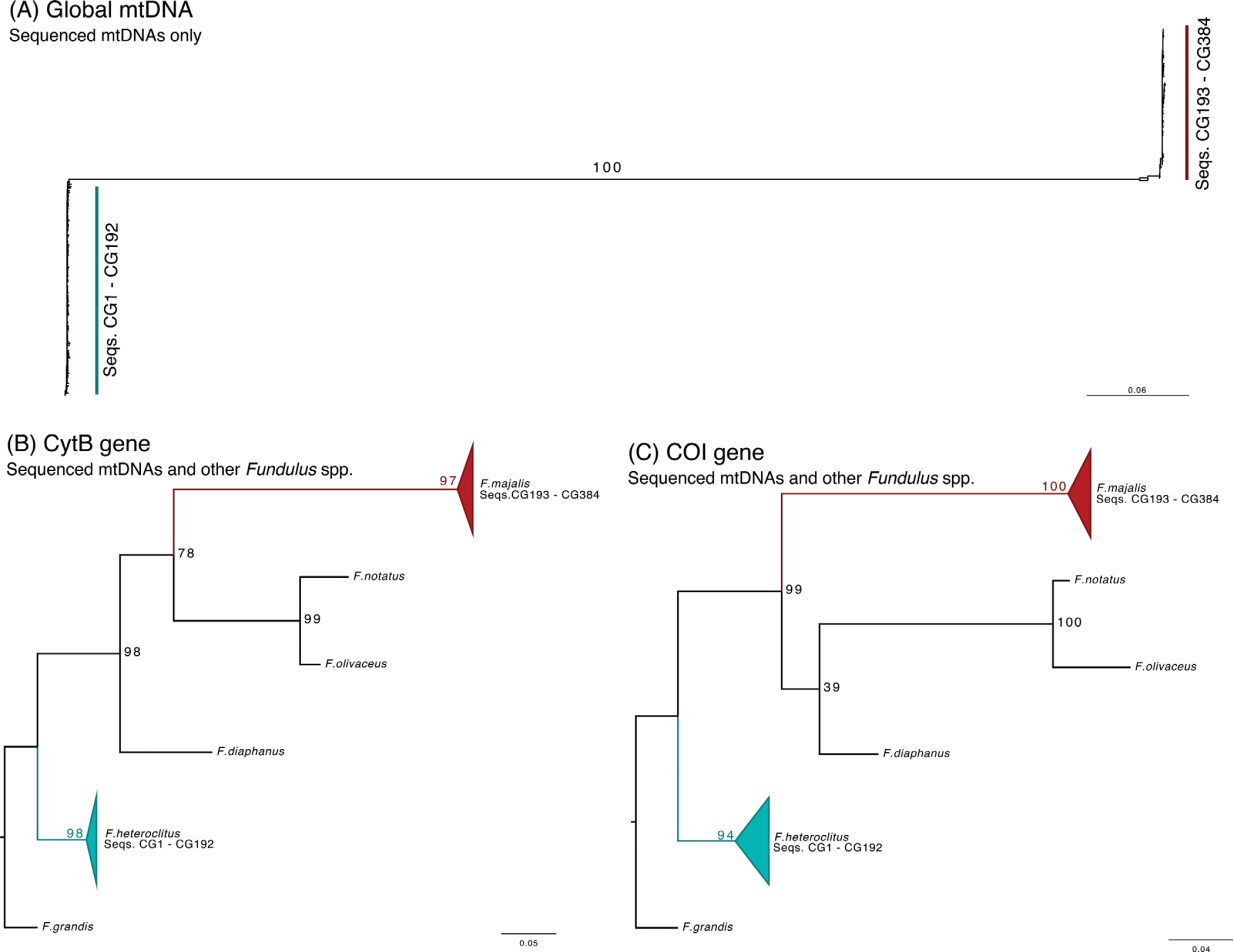
Maximum likelihood phylogenetic reconstructions. A) Global phylogenetic tree produced using 5,088 sites dispersed throughout the entire mtDNA of most samples sequenced in this study. B) Gene tree for the CytB gene. Sequences CG1 through CG192 (*F*. *heteroclitus* samples) clustered with the *F*. *heteroclitus* CytB NCBI sequence (blue cartoon). Similarly, samples CG193 through CG384 (*F*. *majalis* samples) clustered with the *F*. *majalis* CytB NCBI sequence (red cartoon). C) Just as for CytB, sequenced samples form clades with their respective reference COI sequence from NCBI in the case of *F*. *heteroclitus* and from BOLD system v3 in the case of *F*. *majalis*.

Overall, the amount of degenerate loci calls (*i*.*e*. putatively heteroplasmic) was low in both *F*. *heteroclitus* and *F*. *majalis* (Fig 5) with most degenerate calls being R (A/G) and Y (C/T) in both species. We reserve any comment regarding real measures of heteroplasmy in these populations, as it is difficult to accurately differentiate real degenerate calls from PCR or sequencing mistakes. We removed these sites from subsequent analysis. To show that our experiment successfully sequenced individual mtDNAs without cross-species contamination, we conducted phylogenetic inferences with all samples (Fig 6). Unrooted phylogenetic inference conducted only with sequenced samples showed two strongly supported clades (100% bootstrap support) corresponding to *F*. *heteroclitus* samples (CG1 –CG192) and *F*. *majalis* samples (CG193 –CG384; Fig 6A). Two *F*. *majalis* sequences, CG 257 and CG 317, were observed outside of the major *F*. *majalis* clade. Both of these sequences show large number of unique base calls to each sequence, *i*.*e*. not seen as a major or minor allele in either the other *majalis* or *heteroclitus*. These sequences are likely outliers of the experiment. For future studies using this data, we recommend the filtering of these 2 sequences. We also conducted a phylogenetic inference using publically available data from the COI and CytB genes. We incorporated a small number of *Fundulus* species including reference sequences for the genes of *F*. *heteroclitus* and *F*. *majalis*. In both cases, all samples of a given species formed well-supported clades with their respective reference sequences. The CytB gene tree (Fig 6B) shows an equivalent topology to previously published species trees ([28]; species tree built with the CytB gene (mtDNA), and the glycosyltransferase and recombination activating gene 1 (nuclear) sequences). The COI gene tree (Fig 6C), on the other hand, shows a weakly supported topology for the *F*. *majalis*, *F*. *diaphanus* and the *F*. *notatus / F*. *olivaceous* clade. This weak phylogenetic signal is likely due to COI’s inability to resolve evolutionarily young species ([50] and references within). To further investigate any signs of cross-species contamination, we attempted to map *F*. *majalis* reads to the *F*. *heteroclitus* reference and vice versa using bowtie2. Both cases resulted in very low alignment statistics: mean mapping efficiency ~ 9.07%, mean coverage ~ 4.416 (sd = 19.2), and mean mapping quality ~ 6.8.

## Discussion

The use of high-throughput sequencing for evolutionary and ecological studies is becoming more common [51, 52]. These approaches include sequencing reduced representative genomic libraries [13, 53], which can be applied to 100s of individuals, or targeting specific portions of the genome [14, 54]. Presented here is a simple PCR approach to sequence 383 complete and nearly complete mitochondrial genomes, half from a species with no reference genome. Other similar approaches either enrich for mitochondrial genomes using capture probes [14, 55] or simply sequence genomic libraries at low depth and use bioinformatics to identify the mitochondrial genome [56]. Compared to the mitochondrial sequencing approach described here, these other approaches sequence far fewer individuals, require more steps, reagents, kits and specialized equipment, and consequently can be more expensive.

### Technical Considerations of our Approach

Our approach provides sufficient coverage to sequence complete mitochondrial genomes in hundreds of individuals and the ability to produce a *de novo* complete genome. This approach is cost-effective (~$1.65/sample, Table 2) because it uses small reaction volumes to minimize reagent costs and unmodified (non-phosphorylated) barcoded adaptors. Rather than phosphorylated adaptors, which are expensive to synthesize, the protocol relies on an end-repair step to make unmodified adaptors suitable for ligation. While this approach is likely less efficient than using adaptors with a synthesized 5'phosphate (*i*.*e*. may result in lower ligation efficiencies), the sequencing capacity of the Illlumina 2500 platform provides sufficient coverage to render this inefficiency moot. The protocol uses the same adaptor strategy as that used in GBS [13]. This strategy relies on a barcoded adaptor ligating to one end of each DNA fragment and a common adaptor ligating to the other end of the DNA fragment (thereby obviating the need to synthesize two barcoded adaptors for each sample). Although a barcoded adaptor ligating to one end of each DNA fragment and a common adaptor ligating to the other end is not the only possible ligation combination (*e*.*g*., the barcoded or common adaptor could be ligated to both ends of the same piece of DNA), it is the only combination that will sequence on the Illumina platform. The final library preparation step is a PCR step using one primer complementary to the non-barcoded portion of the barcoded adaptor and another primer complementary to the common adaptor. Surprisingly, although ~1/2 of the ligation products should have either only the barcoded adaptor or only the common adaptor, these products do not amplify well because of hairpin structure formation [54]. Thus, when only one primer is added to the PCR reaction, little product is synthesized. In contrast, when both primers are added to the PCR reaction, synthesized product can be visualized on an agarose gel.

As well as being cost-effective with respect to reagents, this approach is also easily accomplished. One person can easily complete the protocol, which takes two days due to an overnight ligation step. However, it could be shortened to one day with the use of a rapid ligation step, which also would increase cost. Further, the only specialized equipment needed is a 96-well sample PCR machine and multichannel pipette, which are standard equipment in most molecular biology laboratories.

We obtained mtDNA sequences from our samples using overlapping PCR amplifications due to the simplicity and low cost of this approach. Our primers were designed based on an aliment of four fish species, including *F*. *heteroclitus*, but not *F*. *majalis*, and the primers worked equally well on both *F*. *heteroclitus* and *F*. *majalis*. However, with other novel species, one might need to test various primer pairs. Alternatively, there are other methods available to obtain *de novo* mitochondrial genomes. These methods include, but are not limited to, reconstructing mitochondrial genomes directly from genomic shot-gun sequencing [56], long range PCR amplifications [57], chips or beads for capturing mtDNAs [55, 58–60], or approaches seeking to isolate full organelles followed by mtDNA isolation [61, 62]. These latter two approaches are especially useful when seeking rare mitochondrial variants, which might be confounded with PCR error. Our results show that the primers used for *F*. *heteroclitus* produce amplicons that represent the complete mitochondrial genome with the added benefit of effectively avoiding mitochondrial nuclear encoded pseudo-genes and non functional mitochondrial fragments (*numts* [63]). Mapping barcode specific reads to a reference genome results in the assembly of good quality mitochondrial sequences for each sample. It should also be noted that this approach would work equally well with other organelle genomes, plastids, plasmids or bacterial genomes.

### Coverage of Sequenced Products

We sequenced 192 complete *F*. *heteroclitus* mitochondrial genomes with on average, 62-fold coverage. Sample coverage had a large standard deviation (89.7) due to over-representation of the regions at the two extremes of the reference genome, which also affects average coverage. These high coverage regions are found on parts of three different amplicons, and thus the greater sequencing depth is not due to more starting PCR product. The remaining and majority of the genomic regions showed average coverage close to ~30-fold (Fig 3B) for individual alignments. Furthermore, most individuals had high mapping quality (75% of the individuals had an mQ > 41, Fig 3E), and 107 out of 192 individuals had 100% non-N sequences (Fig 3C). This coverage depth and mapping quality is sufficient for determining robust, individual mitochondrial sequences that can be used in most downstream analyses.

We also sequenced 191 *F*. *majalis* mitochondrial genomes. To map individual reads, we assembled a *de novo F*. *majalis* mitochondrial genome. Our results with *F*. *majalis* demonstrate that while having a well-annotated reference genome is convenient, it is not necessary for these types of studies. In fact, since most genetic changes occurring in the mitochondrion are base substitutions, very few genetic changes result in drastic length alterations [9]. This allows assemblies to be robust and easy when the contigs generated *de novo* are compared to closely related taxa through BLAST hits or other databases containing genic data.

The *F*. *majalis* reference genome was built *de novo* from raw reads using a bait and iteration approach. These assemblies were done in two independent runs using two bait sequences available in NCBI the CytB Gene sequence of *F*. *majalis* and the full mtDNA of *F*. *heteroclitus*. These two assemblies had two key differences; the first is the time to completion (*F*. *heteroclitus* mtDNA = 27 iterations, CytB = 105 iterations), and the second is the length of the final contig (CytB = 9,763, *F*. *heteroclitus* mtDNA = 16,812). The MITObim algorithm is sensible to the genetic distance between the bait and the read pool [40]. However, since both *F*. *heteroclitus* and *F*. *majalis* are evolutionarily close, the differences here primarily reflect the original bait length. The problematic region in both *F*. *majalis* assemblies seems to be a product of the read underrepresentation (see below). This seems apparent, as the *de novo F*. *heteroclitus* assembly had no problem in generating a complete mtDNA even though it started using the same CytB Gene region as bait.

The undefined region in the *F*. *majalis* samples and *de novo* reference is puzzling. Different contigs obtained through *de novo* assembly using SPAdes on all available reads resulted in contigs covering all areas of the genome, including the low coverage region (verified *via* BLAST hits). For the low coverage region, when pooled reads were mapped back to the consensus *F*. *majalis* sequence, the resulting sequence achieves coverage depth of approximately ~300-fold across all individuals. This coverage level is low compared to the rest of the genome; however, at face value, it suggests that the reads are not missing but rather are at low counts (*i*.*e*., underrepresented).

The low coverage *F*. *majalis* mitochondrial region maps to amplicon 3 (Fig 4A, Table 1), suggesting that this amplification product did not sequence well. Because PCR amplicons were pooled and then processed together for Illumina sequencing, it is difficult to explain why this region would sequence less well than the other amplification products, which make up the remaining 75% of the genome. Further, because this low coverage region contains essential genes involved in a crucial metabolic pathway (a fragment from ATP synthase subunit 8 (ATP 8) gene, the ATP synthase subunit 6 gene (ATP 6), the cytochrome oxidase subunit 3 (CO III) gene, and 3 subunits of the NADH dehydrogenase complex (ND 3, ND 4, and ND 4L), all genes involved in the oxidative phosphorylation pathway), low coverage is unlikely to be due to a complete fragment deletion. We also doubt that the low coverage is the result of bad primer products since the fragments sequenced for this region were obtained using primers that were also used in *F*. *heteroclitus* and were confirmed to work on *F*. *majalis* through Sanger sequencing. Instead, this low coverage is likely related to a technical (human) error that occurred prior to library construction, for example, using the wrong plate of amplification products.

Both species had similar but not identical regions where the sequence depth *per* individual was extraordinarily high (Figs 3A and 4A). For *F*. *heteroclitus*, overrepresented regions spanned portions of the cytochrome B gene, the D-loop, the 12S ribosomal gene, and portions of the 16S ribosomal gene. Coverage in these regions showed three distinctive peaks with a trough in the D-loop region (Fig 3A). For *F*. *majalis*, high coverage only spanned a region including the D-loop, the 12S ribosomal gene, and portions of the 16S ribosomal gene (Fig 4A). While the overrepresented regions correspond to amplicon 1b in *F*. *majalis* (Fig 4A), this is not the case in *F*. *heteroclitus* (Fig 3A), where the overrepresented regions are partially shared among three amplicons. Studies have documented potential causes that may result in regions being overrepresented (see [64]). GC content, for instance, is a common source of bias in sequencing-by-synthesis [65, 66]. However, our results show that the overrepresented regions have no particular correlation with GC content. Since both species were sequenced in the same run and assuming that the overrepresentation of these reads was caused by similar factors, it is unlikely that the high coverage was related to PCR amplification bias or primer performance with the mitochondrial templates. However, the overrepresentation could be related to PCR amplification bias during library construction, which can depend on base composition and could be minimized by modifying PCR conditions [67].

The overrepresented regions also showed a trough in the D-loop, which could be related to overlapping primer regions. Other studies mapping reads to a reference from overlapping mitochondrial amplicons noted that coverage depth tends to fluctuate at regions where primers overlap [68]. While this observation could explain the trough seen in the D-loop of *F*. *heteroclitus* reads, we did not observe any other *drastic* coverage fluctuations associated with overlapping amplicons. Additionally, the trough observed in the D-loop could also be explained by the presence of a G followed by a repetitive region of 15 C bases (position 15,679 in *F*. *heteroclitus* reference mtDNA; GenBank: FJ445403.1). C and G nucleotides tend to have higher error rates than A or T, and coverage has been shown to decrease drastically in the neighboring regions of error prone motifs [64]. While these uneven coverage regions may represent suboptimal coverage potential of the sequencing run, it ultimately does not subtract from the overall cost-efficiency of our approach as the final genomes have sufficient overall coverage.

### Read Quality and Optimization Considerations

Approximately six million reads passed our filtering; however, only ~75% mapped to the reference mitochondrial genome. Non-mapping reads are not nuclear DNA contamination because they do not map to the *F*. *heteroclitus* nuclear genome either. Nor do they appear to be contamination because contigs made from the non-mapping reads in a BLASTn search only match *Fundulus* taxa mitochondrial genomes if they match anything at all. Instead, they likely are mitochondrial sequences that do not map to the reference mitochondrial genomes due to low phred quality (Fig 2E and 2F) and resulting sequencing errors, which make alignments fail.

Potential sequence errors could have been introduced at the initial PCR step since we chose to amplify our samples in long (>1000 bp) amplicons using a traditional Taq polymerase without proofreading capabilities. While this problem could be tackled through the use of high fidelity polymerases with proofreading capabilities, such reagents may ramp up the overall cost of the experiment. It is important to note that, for the library sequenced in this experiment, only 54% of clusters passed filter. This is unique to our sequencing run, thus future replicates of our approach may see an overall increase of coverage. According to Illumina support (http://support.illumina.com/help/SequencingAnalysisWorkflow/Content/Vault/Informatics/Sequencing_Analysis/CASAVA/swSEQ_mCA_PercentageofClustersP.htm), very few clusters passing filter can be due to many factors including: a poor flow cell, unblocked DNA, faint clusters, out of focus clusters, poor matrix, a fluidics or sequencing failure, bubbles in individual tiles, too many clusters, large clusters, and high phasing or pre-phasing. If the problem is isolated to early sequencing cycles, then filtering might throw away very good data. Additionally, early cycles are fairly resistant to minor focus and fluidics problems. Because the sequencing facility could not explain why only 57% of sequence reads passed filter, we analyzed all data. Given the similar percentage of our reads that failed to map, we suspect that many of the unmapped reads would not have passed filter.

Reads showed a surprising *per*-base quality score distribution. In all reads, both 5’ and 3’ ends show good phred scores while displaying lower scores in the read center. The quality trimming we used occurs from the 3' and 5' ends of the reads until a threshold is met. Even though mapping reads display this low phred score in the center, the rest of the read has consistent high phred scores throughout (Fig 2C and 2D). Non-mapping reads also display interior low phred quality scores; however there is high variation in quality scores throughout the rest of the read (Fig 2E and 2F). Based on these observations, we believe that many of these non-mapping reads should have been filtered out; however the flanking high quality scores prevented such filtering. A bioinformatics solution to this issue is to add another, more stringent filtering step, which only keeps reads for which a high percentage (could be 100%) of the base pairs within a read have a minimal phred score. We have included this additional, optional step in S3 File. This step should not be necessary when only reads that pass filter are used.

### Mitochondrial DNA as a marker for population studies

While the mitochondrial genome is a widely used marker for population genetic studies, it is not without drawbacks. Each locus within the molecule evolves at different rates with different functional constrains, and thus the use of single genes for population genetics or phylogenetic analysis may suffer from confounding effects. One of the underlying motivations behind this and other pipelines aimed at reconstructing full mitochondrial genomes is the generation of datasets capturing comprehensive evolutionary signals. It is also important to note that as a non-recombinant molecule, this marker only provides access to a single locus (37 linked loci in the case of animal mtDNA). As such, there are inherent limitations for population genetic studies. Bazin *et*. *al*. [69], for instance, argued that measures of effective population sizes (N_e_) are not properly captured in mtDNA variation, instead arguing for the use of neutral markers as accurate estimators. While other studies have debated the Bazin *et al*. argument (see [70, 71]), it is clear that the inclusion of nuclear markers can only enhance the evolutionary signals captured in datasets. The approach discussed in this paper can easily be extended to include nuclear markers through the addition of PCR amplified nuclear loci. These fragments can be uniquely barcoded and sequenced in the same lane as the mtDNAs without major coverage loss for any marker.

## Conclusion

In conclusion, we present a cost-effective approach to prepare sequencing libraries for whole mitochondrial genomes for many individuals. Our strategy uses small reaction volumes and unmodified (non-phosphorylated) barcoded adaptors to minimize reagent costs. Our approach is optimized for sequencing whole mitochondrial genomes on Illumina platforms. However, with the proper modifications, this approach could be applied to other sequencing platforms or other types of genomes. We demonstrated our approach by sequencing 383 mitochondrial sequences from *Fundulus heteroclitus* and *Fundulus majalis*. We successfully amplified and sequenced the mitochondrial genomes of these two species with high coverage levels. A reference genome available in NCBI was used as alignment target for *F*. *heteroclitus* samples. However, for *F*. *majalis*, we assembled a genome *de novo* as target for alignment, thus showing that a reference genome is not a necessity for these kinds of experiments.

## Supporting Information

S1 File Extended Protocol for mtDNA library Preparation. Detail protocol for mtDNA library preparations.
(DOCX)

S2 File Individual Reads SRR numbers. A spreadsheet file containing combined metadata from the SRA submission. The file details the barcode ID of all samples with their respective SRR numbers for retrieval in the NCBI SRA database.
(XLSX)

S3 File Bioinformatics Pipeline. Detailed pipeline for the assembly for individual mtDNAs from read data.
(DOCX)
