# Supplementary material for: A Cost-Effective Approach to Sequence Hundreds of Complete Mitochondrial Genomes
Source: PLoS One. 2016 Aug 9;11(8):e0160958. doi: 10.1371/journal.pone.0160958 (PMC4978415; doi:10.1371/journal.pone.0160958)
Supplement: S1 File — Detail protocol for mtDNA library preparations. (DOCX) [file pone.0160958.s001.docx]

# Mitochondrial DNA Sequencing

Start with 100 ng of PCR amplified mitochondrial DNA dried down (If mtDNA was obtained though PCR amplification, start with 100 ng of dried down PCR amplified mtDNA).

Reconstitute in 2 ul of H_2_O overnight. (Cover with tape. Next day spin down before removing tape.)

(Note: 0.04 pmol of 300 bp DNA ~= 7.9 ng; this is ~10x less than adaptor concentration).

**Fragment Mitochondrial DNAs:**

**Incubators needed:**

- 37º C thermal cycler. Pre-heat PCR machine to 37º C. Program for 30 minutes at 37º C followed by 4º C soak. Before use, VORTEX NEBNext dsDNA Fragmentase for 3 seconds, quick spin and place on ice.

Make Fragmentase Master Mix on ice, in order listed.

| Total Amount | Amount | Reagent | 10 Reactions | 100 Reactions | XX  Reactions |
| --- | --- | --- | --- | --- | --- |
|  | 2 ul | H_2_O | 30 | 300 |  |
|  | 0.5 ul | 10x fragmentation buffer v2 | 10 | 100 |  |
| 20-30 units | 0.5 ul | Fragmentase | 10 | 100 |  |
| TOTAL | 3 ul |  | 60 | 600 |  |

Add 3 ul fragmentase Master Mix to each 2 ul of sample. Mix well by pipetting up and down 8-10 times (NEB recommends vortexing if possible).

Cover with tape and place in PCR machine: 37º for 30 minutes followed by 4º C soak.

Prepare mix of 8 ul of 500 mM EDTA and 32 ul of 10 mM Tris and add 47 ul to sample

| Total Amount | Amount | Reagent | 10 Reactions | 100 Reactions | XX  Reactions |
| --- | --- | --- | --- | --- | --- |
|  | 10 ul | 500 mM EDTA | 80 | 800 |  |
|  | 40 ul | 10 mM Tris | 320 | 3200 |  |
| TOTAL | 50 ul |  | 400 | 4000 |  |

**Size Select and Elute**

**Want sizes < 300 bp** (fragments <300 bp remain in supernatant): Add 0.55x (27.5 ul) of beads (20% PEG 8000, 2.5 M NaCl). Bind 10 minutes. Magnetize and **keep supernatant**.

Add same volume of beads (27.5 ul) to supernatant. Bind 10 minutes. Magnetize. Discard super. Wash 2x with 70% EtOH. Air dry 2-5 minutes.

Note: when start with 100 ng of DNA and digest for 30 minutes or 500 ng and digest for 20 minutes, recover ~10% (10 ng or 50 ng, respectively).

**Add Barcoded adaptors to each sample**

Add 4 ul of 100 nM barcoded adaptors to each sample, directly to beads. The barcoded adaptors are 100 nM each of the barcoded and common adaptor.

**End Repair**

**Incubators needed:**

- 20º C thermal cycler. Pre-heat PCR machine to 20º C. Program for 30 minutes at 20º C followed by 20 minutes at 75º C to heat kill the enzymes.

End Repair Master Mix.

| Total Amount | Amount | Reagent | 10 Reactions | 100 Reactions | XX  Reactions |
| --- | --- | --- | --- | --- | --- |
|  | 0.5 ul | H_2_O | 90 | 900 |  |
|  | 1.0 ul | 10x ligation buffer | 12.5 | 125 |  |
|  | 4.0 ul | 1 mM dNTP mix | 12.5 | 125 |  |
| 5 U | 0.25 ul | T4 polynucleotide kinase | 2.5 | 25 |  |
| 1.5 U | 0.25 ul | T4 DNA polymerase | 2.5 | 25 |  |
| TOTAL | 6.0 ul |  | 125 | 1250 |  |

Add 6 ul End Repair Master Mix to beads. Incubate at 20º C for 30 minutes. Heat kill polymerase at 75º C for 20 minutes. Quick spin if needed.

**Barcoded Adaptor Ligation**

**Incubators needed:**

- 16º C thermal cycler.

Ligation Master Mix

| Total Amount | Amount | Reagent | 10 Reactions | 100 Reactions | XX Reactions |
| --- | --- | --- | --- | --- | --- |
|  | 2.45 ul | H_2_O | 25 | 250 |  |
|  | 0.3 ul | 10x ligation buffer | 67.5 | 675 |  |
|  | 0.25 ul | T4 DNA ligase | 2.5 | 25 |  |
| Total | 3.0 ul |  | 95 | 950 |  |

Add 3 ul Ligation Master Mix to each sample. Incubate at 16º C overnight (12-16 hours). Increase volume to 40 ul with 27 ul TE. Add 40 ul of PEG/salt (20% PEG 8000, 2.5 M NaCl), bind, wash with 70% EtOH 2x, dry 2-5 minutes, and elute in 30 ul of 0.1x TE.

**Pooling and Clean Up**

Pool 5-20 ul from each well and bead purify a portion for PCR template. Want to concentrate the pool 10-fold.

Add 500 ul of carboxyl beads in 20% PEG 8000, 2.5 M NaCl to 500 ul of pooled, ligated, DNAs. Let bind to beads for 5-10 minutes.

Magnetize.

Remove supernatant.

Wash beads 2x with 200 ul 70% EtOH.

Let air dry 3-5 minutes.

Elute with 50 ul of 10 mM Tris, 5 minutes.

Magnetize and save eluate in clean 1.5 ml tube.

# PCR Amplification

| **Reagent** | **Volume (µL)** |
| --- | --- |
| DNA from previous step | 1 |
| NEB 2x Taq Master Mix | 25 |
| PCR Primer Mix (10 pmol/µL each primer) | 2 |
| H_2_O | 22 |
| **Total** | **50** |

PCR Primers

5'AATGATACGGCGACCACCGAGATCTACACTCTTTCCCTACACGACGCTCTTCCGATCT 5'CAAGCAGAAGACGGCATACGAGATCGGTCTCGGCATTCCTGCTGAACCGCTCTTCCGATCT

- Amplify using the following PCR cycling protocol:

a. 30 seconds at 94°C

b. 15-22 (variable) cycles of:

10 seconds at 98°C

30 seconds at 65°C

30 seconds at 72°C

c. 5 minutes at 72°C

d. Hold at 4°C

## Clean-up

- For each of the PCR reactions, purify with 0.7x beads (35 ul).
- Elute in 25 µL 10 mM Tris.

## Evaluate libraries

Run sample on Bioanalyzer according to manufacturer’s instructions. Alternatively, run on gel.
