# Supplementary material for: A Cost-Effective Approach to Sequence Hundreds of Complete Mitochondrial Genomes
Source: PLoS One. 2016 Aug 9;11(8):e0160958. doi: 10.1371/journal.pone.0160958 (PMC4978415; doi:10.1371/journal.pone.0160958)
Supplement: S3 File — Detailed pipeline for the assembly for individual mtDNAs from read data. (DOCX) [file pone.0160958.s003.docx]

**Example mtDNA ‘Raw Read-to-Consensus’ Pipeline**

This example pipeline covers the basic steps of a bioinformatics pipeline used to process raw illumina reads into consensus fasta/fastq sequences corresponding to each individual barcoded sample. This example is envisioned for anyone intending to replicate the experiment presented in this manuscript using free and publicly available bioinformatics tools. We recognize that there are other -more sophisticated- methods for processing the data from raw reads to consensus sequence. Namely, those consisting of custom made python, perl, C, or other programming languages scripts. For this example we assume the reader has some basic understanding of bash scripting and using the command line. Notice that since this pipeline mainly consists of free and publicly available software, all extended information about the individual capabilities of the used software is available in their own individual documentation.

**Software required to run the pipeline:**

trim_galore v0.3.7: <http://www.bioinformatics.babraham.ac.uk/projects/trim_galore/>

fastx_toolkit v0.0.13: <http://hannonlab.cshl.edu/fastx_toolkit/commandline.html>

FastQC v0.11.4: <http://www.bioinformatics.babraham.ac.uk/projects/fastqc/>

mito_barcode_splitter.sh: (custom script for the barcodes used in this study; it uses fastx_barcode_splitter.pl from fastx_toolkit)

cutadapt v1.9: <https://cutadapt.readthedocs.org/en/stable/>

bowtie2 v2.2.3: <http://bowtie-bio.sourceforge.net/bowtie2/index.shtml>

samtools v1.2: <http://samtools.sourceforge.net>

bcftools v1.2: <http://samtools.github.io/bcftools/>

vcfutilis.pl: <https://github.com/lh3/samtools/blob/master/bcftools/vcfutils.pl> (Download as a script with the same name ‘vcfutils.pl’ and make a soft link to your path)

seqtk v1.0: <https://github.com/lh3/seqtk>

For this example pipeline to work all of these software must be installed and configured correctly in your computer.

Please note that the symbol ./ denotes the current working folder. For this pipeline, such folder is likely to be the folder containing the reads to be processed. Otherwise, please modify the code accordingly.

**1. First quality filter:** Trim low Phred score sequences (< 20 in this example) and trim off adaptors, and get rid of reads smaller than an arbitrary threshold length (50 bp in this example).

trim_galore -q 20 -a AGATCGGAAGAGC --length 50 ./my_reads_in_fastq.fastq

-q: Phred Score threshold

--length: minimum length threshold

It sometimes occurs that adaptors ligate to each other and are sequenced (i.e. adaptors are sequenced as part the insert). This will result in short sequences of varying qualities that will infiltrate the read pools of their corresponding barcodes. A second trim_galore step should eliminate these artifacts

trim_galore -q 20 -a GCTCTTCCGATCT --length 50 ./my_reads_in_fastq.fastq

At any point throughout this pipeline you may check the quality of the raw reads by feeding the fastq files to the program **FastQC.**

Alternatively, if your adaptor has an overhang, then remove the 2 bp overhang with the adaptors

trim_galore -q 20 -a NNAGATCGGAAGAGC --length 50 ./my_reads_in_fastq.fastq

**2. Second quality filter (Optional):** Filter reads with per base quality score less than a threshold (20 in this example). This is a very stringent optional step that will ensure the output of only good quality reads. This step, however, may result in a drastic reduction in the number of usable reads depending on the value assigned to the parameter –p (see below).

fastq_quality_filter –v -Q33 -q 20 -p 100 -i ./my_reads_in_fastq.fastq -o ./my_reads_in_fastq_trimmed.fastq

-v: Verbose option

-Q33: Refer to the encoding of the reads (Phred+33)

-p: Percent of reads with the threshold quality score declared in the –q option

-i: In-file

-o: Out-file

**3. Sort by barcodes:** Run the barcode splitter script in the folder containing my_reads_in_fastq.fastq. This script sequentially removes barcodes of different sizes, from 11mers to 6mers. Modify the path and folder names as appropriate for your computer. The barcode files (e.g., 6mer.txt) are tab delimited text files with the first column the barcode name and the second column the barcode sequence. For example,

#6mer

CG1 AACTCG

CG2 CCAGCG

CG3 TTGACG

CG4 GGTACG

CG5 ATTGCG

CG6 CGGTCG

CG7 TGCGCG

CG8 GTATCG

CG9 AACGCG

CG10 CCTACG

CG11 TTACCG

CG12 AGGCCG

CG13 GCAGCG

CG14 TAGACG

#! /bin/bash

#To run this script, type mito_barcode_splitter.sh while in the Mito-Lib_fastq folder

#Since barcodes are not the same size, need to sort sequentially, starting with the longest #barcodes, allowing no mismatches. The input is the unmatched output of the previous #barcode.

#The barcode files are stored as .txt files in Mito-Lib_fastq/

#For the first line, need to input the appropriate trimmed file and pipe (|) it to #fastx_barcode_splitter.pl

cat my_reads_trimmed.fq | /Users/username/programs/fastx_barcode_splitter.pl --bcfile /Users/username/Mito-Lib_fastq/11mer.txt --bol --mismatches 0 --prefix /Users/username/Mito-Lib_fastq/split_samples/sample_11mer_ --suffix ".fq"

cat /Users/ username /Mito-Lib_fastq/split_samples/sample_11mer_unmatched.fq | /Users/username/programs/fastx_barcode_splitter.pl --bcfile /Users/username/Mito-Lib_fastq/split_samples/sample_10mer_ --suffix ".fq"

cat /Users/username/Mito-Lib_fastq/split_samples/sample_10mer_unmatched.fq | /Users/username/programs/fastx_barcode_splitter.pl --bcfile /Users/username/Mito-Lib_fastq/9mer.txt --bol --mismatches 0 --prefix /Users/username/Mito-Lib_fastq/split_samples/sample_9mer_ --suffix ".fq"

cat /Users/username/Mito-Lib_fastq/split_samples/sample_9mer_unmatched.fq | /Users/username/programs/fastx_barcode_splitter.pl --bcfile /Users/username/Mito-Lib_fastq/8mer.txt --bol --mismatches 0 --prefix /Users/username/Mito-Lib_fastq/split_samples/sample_8mer_ --suffix ".fq"

cat /Users/username/Mito-Lib_fastq/split_samples/sample_8mer_unmatched.fq | /Users/username/programs/fastx_barcode_splitter.pl --bcfile /Users/username/Mito-Lib_fastq/7mer.txt --bol --mismatches 0 --prefix /Users/username/Mito-Lib_fastq/split_samples/sample_7mer_ --suffix ".fq"

cat /Users/username/Mito-Lib_fastq/split_samples/sample_7mer_unmatched.fq | /Users/username/programs/fastx_barcode_splitter.pl --bcfile /Users/username/Mito-Lib_fastq/6mer.txt --bol --mismatches 0 --prefix /Users/username/Mito-Lib_fastq/split_samples/sample_6mer_ --suffix ".fq"

To execute type:

mito_barcode_splitter.sh

The script will generate barcode sorted files.

**4. Trim barcode adaptors from barcoded samples:** To keep track of files, we recommend storing all the files generated in this step in an individual folder. We are calling such folder ‘trimmed_barcodes’.

mkdir ./trimmed_barcodes/

cutadapt –m 50 –g ^<barcode> -o ./barcode_ID.fq sample_Nmer_barcodeID.fq

Example*:

cutadapt -m 50 -g ^AACTCG -o ./trimmed_barcodes/CG1.fq sample_6mer_CG1.fq

The notation ‘-g ^’ denotes a 5’ anchored barcode where the start of the read is the 5' end (the barcode is on the 5' or start of the read).

-m: denotes the minimum read size to keep.

*For examples of each individual barcode ID, see Appendix 1.

We now want to trim primers from the sequences. We'll make a new directory for these sequences:

mkdir ./trimmed_barcodes_primers/

Cutadapt can take multiple sequences to trim, but only the best matching adapter will be trimmed from each read. You only want to trim primers from the ends of the reads (not within the sequences). Thus, use anchored 5' and 3' options (-g ^ XXX and -a XXX$, respectively). Also, you need to enter both the primer sequence and the reverse complement of the primer. For example, to remove the primer GATGTGCGATG

cutadapt -m 50 -g ^ GATGTGCGATG -a GATGTGCGATG$ -g CATCGCACATC -a CATCGCACATC$ -o ./trimmed_barcode_primers/CG1.fq sample_6mer_CG1.fq

This step will generate individual files (named after each barcode) with trimmed sequences ready to be aligned to a reference or used in *de novo* assembly. This example protocol will only cover the former. Please consult the SPAdes documentation for *de novo* assembly that can then be used as a reference for alignment.

**5. Alignment with Bowtie2:**

5.1: Build an index file for Bowtie2 to use during the alignment:

bowtie2-build -f <Reference_genome.fasta> <Basename_of_index*>

*The term “Basename_of_index” refers to a name given to the indexed reference. This term will be used during the alignment as one of the main arguments of Bowtie2 (see below).

5.2: To keep track of input/output files we recommend creating the following sets of folders: sam_files, bam_files, sorted_bam, consensus, and consensus_fasta.

mkdir ./sam_files

mkdir ./bam_files

mkdir ./sorted_bam

mkdir ./consensus

mkdir ./consensus_fasta

5.3: Proceed to align reads with Bowtie2. This will generate a SAM file as output

bowtie2 -x <Basename_of_index> ./ trimmed_barcode_primers/<sample_Nmer_barcodeID.fq> –S. /sam_files/<Nmer_barcodeID.sam>

To effectively process all files at once we recommend using a basic loop approach:

#Change directory to the folder containing all trimmed barcodes

cd ./Trimmed_barcodes

files=(*)

#Perform the loop

for i in "${files[@]}"

do bowtie2 -x <Basename_of_index> ./ trimmed_barcode_primers/$i -S ./sam_files/$i.sam

done

5.4: Use samtools to convert the SAM files to BAM files.

samtools view –bS sam_files/<sample_Nmer_barcodeID.sam> > bam_files/ <sample_Nmer_barcodeID.bam>

The loop approach:

for i in "${files[@]}"

do samtools view -bS ./sam_files/$i.sam > ./bam_files/$i.bam

done

5.5: Use samtools to index the reference genome

samtools faidx <Reference_genome.fasta>

5.6: Use samtools to sort the BAM files

samtools sort ./bam_files/<sample_Nmer_barcodeID.bam> ./sorted_bam/ <sample_Nmer_barcodeID>

Or

for i in "${files[@]}"

do samtools sort ./bam_files/$i.bam ./sorted_bam/$i

done

5.7: Use samtools to index the BAM files

samtools index ./sorted_bam/ <sample_Nmer_barcodeID>

Or

for i in "${files[@]}"

do samtools index ./sorted_bam/$i.bam

done

5.8: Use samtools, bcftools and vcfutilis to generate a consensus fastq file

samtools mpileup -uf <Reference_Genome.fasta> ./sorted_bam/ <sample_Nmer_barcodeID.bam> | bcftools call -c | vcfutils.pl vcf2fq > ./consensus/ <sample_Nmer_barcodeID>

Or

for i in "${files[@]}"

do samtools mpileup -uf <Reference_Genome.fasta> ./sorted_bam/$i.bam | bcftools call -c | vcfutils.pl vcf2fq > ./consensus/$i

done

For this step the reference genome used must be the same one indexed in step 5.5. Also the script vcfutilis must be softlinked to the path in order to be called as described. In case this doesn’t work, it is possible to call the script by its full path ‘./ vcfutils.pl.’

5.9: Use seqtk to convert the consensus fastq files into fasta files.

seqtk seq -a ./consensus/<sample_Nmer_barcodeID> > ./consensus_fasta/< sample_Nmer_barcodeID.fasta>

Or

for i in "${files[@]}"

do seqtk seq -a ./consensus/$i > ./consensus_fasta/$i.fasta

done

This should result in fasta files containing individual consensus DNA sequences.

**Appendix 1:Example code for all barcode (GC and TA) sequences trimming procedure (Step 4):**

cutadapt -m 50 -g ^AACTCG -o ./trimmed_barcode/CG1.fq sample_6mer_CG1.fq

cutadapt -m 50 -g ^CCAGCG -o ./trimmed_barcode/CG2.fq sample_6mer_CG2.fq

cutadapt -m 50 -g ^TTGACG -o ./trimmed_barcode/CG3.fq sample_6mer_CG3.fq

cutadapt -m 50 -g ^GGTACG -o ./trimmed_barcode/CG4.fq sample_6mer_CG4.fq

cutadapt -m 50 -g ^ATTGCG -o ./trimmed_barcode/CG5.fq sample_6mer_CG5.fq

cutadapt -m 50 -g ^CGGTCG -o ./trimmed_barcode/CG6.fq sample_6mer_CG6.fq

cutadapt -m 50 -g ^TGCGCG -o ./trimmed_barcode/CG7.fq sample_6mer_CG7.fq

cutadapt -m 50 -g ^GTATCG -o ./trimmed_barcode/CG8.fq sample_6mer_CG8.fq

cutadapt -m 50 -g ^AACGCG -o ./trimmed_barcode/CG193.fq sample_6mer_CG193.fq

cutadapt -m 50 -g ^CCTACG -o ./trimmed_barcode/CG194.fq sample_6mer_CG194.fq

cutadapt -m 50 -g ^TTACCG -o ./trimmed_barcode/CG195.fq sample_6mer_CG195.fq

cutadapt -m 50 -g ^AGGCCG -o ./trimmed_barcode/CG196.fq sample_6mer_CG196.fq

cutadapt -m 50 -g ^GCAGCG -o ./trimmed_barcode/CG197.fq sample_6mer_CG197.fq

cutadapt -m 50 -g ^TAGACG -o ./trimmed_barcode/CG198.fq sample_6mer_CG198.fq

cutadapt -m 50 -g ^GTCACG -o ./trimmed_barcode/CG199.fq sample_6mer_CG199.fq

cutadapt -m 50 -g ^GATCCG -o ./trimmed_barcode/CG200.fq sample_6mer_CG200.fq

cutadapt -m 50 -g ^CTGGCG -o ./trimmed_barcode/CG201.fq sample_6mer_CG201.fq

cutadapt -m 50 -g ^AACTTA -o ./trimmed_barcode/TA1.fq sample_6mer_TA1.fq

cutadapt -m 50 -g ^CCAGTA -o ./trimmed_barcode/TA2.fq sample_6mer_TA2.fq

cutadapt -m 50 -g ^TTGATA -o ./trimmed_barcode/TA3.fq sample_6mer_TA3.fq

cutadapt -m 50 -g ^GGTATA -o ./trimmed_barcode/TA4.fq sample_6mer_TA4.fq

cutadapt -m 50 -g ^ATTGTA -o ./trimmed_barcode/TA5.fq sample_6mer_TA5.fq

cutadapt -m 50 -g ^CGGTTA -o ./trimmed_barcode/TA6.fq sample_6mer_TA6.fq

cutadapt -m 50 -g ^TGCGTA -o ./trimmed_barcode/TA7.fq sample_6mer_TA7.fq

cutadapt -m 50 -g ^GTATTA -o ./trimmed_barcode/TA8.fq sample_6mer_TA8.fq

cutadapt -m 50 -g ^AACGTA -o ./trimmed_barcode/TA193.fq sample_6mer_TA193.fq

cutadapt -m 50 -g ^CCTATA -o ./trimmed_barcode/TA194.fq sample_6mer_TA194.fq

cutadapt -m 50 -g ^TTACTA -o ./trimmed_barcode/TA195.fq sample_6mer_TA195.fq

cutadapt -m 50 -g ^AGGCTA -o ./trimmed_barcode/TA196.fq sample_6mer_TA196.fq

cutadapt -m 50 -g ^GCAGTA -o ./trimmed_barcode/TA197.fq sample_6mer_TA197.fq

cutadapt -m 50 -g ^TAGATA -o ./trimmed_barcode/TA198.fq sample_6mer_TA198.fq

cutadapt -m 50 -g ^GTCATA -o ./trimmed_barcode/TA199.fq sample_6mer_TA199.fq

cutadapt -m 50 -g ^GATCTA -o ./trimmed_barcode/TA200.fq sample_6mer_TA200.fq

cutadapt -m 50 -g ^CTGGTA -o ./trimmed_barcode/TA201.fq sample_6mer_TA201.fq

cutadapt -m 50 -g ^AACCACG -o ./trimmed_barcode/CG9.fq sample_7mer_CG9.fq

cutadapt -m 50 -g ^CCACTCG -o ./trimmed_barcode/CG10.fq sample_7mer_CG10.fq

cutadapt -m 50 -g ^TATAGCG -o ./trimmed_barcode/CG11.fq sample_7mer_CG11.fq

cutadapt -m 50 -g ^GCGCACG -o ./trimmed_barcode/CG12.fq sample_7mer_CG12.fq

cutadapt -m 50 -g ^AATGTCG -o ./trimmed_barcode/CG13.fq sample_7mer_CG13.fq

cutadapt -m 50 -g ^CCGAGCG -o ./trimmed_barcode/CG14.fq sample_7mer_CG14.fq

cutadapt -m 50 -g ^TTACACG -o ./trimmed_barcode/CG15.fq sample_7mer_CG15.fq

cutadapt -m 50 -g ^GGCTTCG -o ./trimmed_barcode/CG16.fq sample_7mer_CG16.fq

cutadapt -m 50 -g ^AAGTGCG -o ./trimmed_barcode/CG17.fq sample_7mer_CG17.fq

cutadapt -m 50 -g ^CCTGACG -o ./trimmed_barcode/CG18.fq sample_7mer_CG18.fq

cutadapt -m 50 -g ^TTCATCG -o ./trimmed_barcode/CG19.fq sample_7mer_CG19.fq

cutadapt -m 50 -g ^GGACGCG -o ./trimmed_barcode/CG20.fq sample_7mer_CG20.fq

cutadapt -m 50 -g ^ACATACG -o ./trimmed_barcode/CG21.fq sample_7mer_CG21.fq

cutadapt -m 50 -g ^CACGGCG -o ./trimmed_barcode/CG22.fq sample_7mer_CG22.fq

cutadapt -m 50 -g ^TGTCTCG -o ./trimmed_barcode/CG23.fq sample_7mer_CG23.fq

cutadapt -m 50 -g ^ATGAACG -o ./trimmed_barcode/CG24.fq sample_7mer_CG24.fq

cutadapt -m 50 -g ^GAGATCG -o ./trimmed_barcode/CG25.fq sample_7mer_CG25.fq

cutadapt -m 50 -g ^CTATGCG -o ./trimmed_barcode/CG26.fq sample_7mer_CG26.fq

cutadapt -m 50 -g ^ACTCGCG -o ./trimmed_barcode/CG27.fq sample_7mer_CG27.fq

cutadapt -m 50 -g ^GTCGACG -o ./trimmed_barcode/CG28.fq sample_7mer_CG28.fq

cutadapt -m 50 -g ^TAATTCG -o ./trimmed_barcode/CG29.fq sample_7mer_CG29.fq

cutadapt -m 50 -g ^CGCAACG -o ./trimmed_barcode/CG30.fq sample_7mer_CG30.fq

cutadapt -m 50 -g ^TCAGGCG -o ./trimmed_barcode/CG31.fq sample_7mer_CG31.fq

cutadapt -m 50 -g ^CTGGTCG -o ./trimmed_barcode/CG32.fq sample_7mer_CG32.fq

cutadapt -m 50 -g ^GATTACG -o ./trimmed_barcode/CG33.fq sample_7mer_CG33.fq

cutadapt -m 50 -g ^AGAATCG -o ./trimmed_barcode/CG34.fq sample_7mer_CG34.fq

cutadapt -m 50 -g ^TAGGACG -o ./trimmed_barcode/CG35.fq sample_7mer_CG35.fq

cutadapt -m 50 -g ^AGATACG -o ./trimmed_barcode/CG202.fq sample_7mer_CG202.fq

cutadapt -m 50 -g ^CCTTCCG -o ./trimmed_barcode/CG203.fq sample_7mer_CG203.fq

cutadapt -m 50 -g ^TGCTGCG -o ./trimmed_barcode/CG204.fq sample_7mer_CG204.fq

cutadapt -m 50 -g ^AACAACG -o ./trimmed_barcode/CG205.fq sample_7mer_CG205.fq

cutadapt -m 50 -g ^CCACGCG -o ./trimmed_barcode/CG206.fq sample_7mer_CG206.fq

cutadapt -m 50 -g ^TTGTCCG -o ./trimmed_barcode/CG207.fq sample_7mer_CG207.fq

cutadapt -m 50 -g ^GGTGACG -o ./trimmed_barcode/CG208.fq sample_7mer_CG208.fq

cutadapt -m 50 -g ^AATCCCG -o ./trimmed_barcode/CG209.fq sample_7mer_CG209.fq

cutadapt -m 50 -g ^CTCAGCG -o ./trimmed_barcode/CG210.fq sample_7mer_CG210.fq

cutadapt -m 50 -g ^TCAGACG -o ./trimmed_barcode/CG211.fq sample_7mer_CG211.fq

cutadapt -m 50 -g ^GAGTGCG -o ./trimmed_barcode/CG212.fq sample_7mer_CG212.fq

cutadapt -m 50 -g ^AGCGCCG -o ./trimmed_barcode/CG213.fq sample_7mer_CG213.fq

cutadapt -m 50 -g ^CCGAACG -o ./trimmed_barcode/CG214.fq sample_7mer_CG214.fq

cutadapt -m 50 -g ^TGACCCG -o ./trimmed_barcode/CG215.fq sample_7mer_CG215.fq

cutadapt -m 50 -g ^GTTCGCG -o ./trimmed_barcode/CG216.fq sample_7mer_CG216.fq

cutadapt -m 50 -g ^TATTACG -o ./trimmed_barcode/CG217.fq sample_7mer_CG217.fq

cutadapt -m 50 -g ^ACAACCG -o ./trimmed_barcode/CG218.fq sample_7mer_CG218.fq

cutadapt -m 50 -g ^GCCGGCG -o ./trimmed_barcode/CG219.fq sample_7mer_CG219.fq

cutadapt -m 50 -g ^GTCTACG -o ./trimmed_barcode/CG220.fq sample_7mer_CG220.fq

cutadapt -m 50 -g ^AGGAGCG -o ./trimmed_barcode/CG221.fq sample_7mer_CG221.fq

cutadapt -m 50 -g ^CAAGCCG -o ./trimmed_barcode/CG222.fq sample_7mer_CG222.fq

cutadapt -m 50 -g ^ATGCACG -o ./trimmed_barcode/CG223.fq sample_7mer_CG223.fq

cutadapt -m 50 -g ^TCTAGCG -o ./trimmed_barcode/CG224.fq sample_7mer_CG224.fq

cutadapt -m 50 -g ^CGCCACG -o ./trimmed_barcode/CG225.fq sample_7mer_CG225.fq

cutadapt -m 50 -g ^ATAGGCG -o ./trimmed_barcode/CG226.fq sample_7mer_CG226.fq

cutadapt -m 50 -g ^GCGCCCG -o ./trimmed_barcode/CG227.fq sample_7mer_CG227.fq

cutadapt -m 50 -g ^GAACACG -o ./trimmed_barcode/CG228.fq sample_7mer_CG228.fq

cutadapt -m 50 -g ^AACCATA -o ./trimmed_barcode/TA9.fq sample_7mer_TA9.fq

cutadapt -m 50 -g ^CCACTTA -o ./trimmed_barcode/TA10.fq sample_7mer_TA10.fq

cutadapt -m 50 -g ^TATAGTA -o ./trimmed_barcode/TA11.fq sample_7mer_TA11.fq

cutadapt -m 50 -g ^GCGCATA -o ./trimmed_barcode/TA12.fq sample_7mer_TA12.fq

cutadapt -m 50 -g ^AATGTTA -o ./trimmed_barcode/TA13.fq sample_7mer_TA13.fq

cutadapt -m 50 -g ^CCGAGTA -o ./trimmed_barcode/TA14.fq sample_7mer_TA14.fq

cutadapt -m 50 -g ^TTACATA -o ./trimmed_barcode/TA15.fq sample_7mer_TA15.fq

cutadapt -m 50 -g ^GGCTTTA -o ./trimmed_barcode/TA16.fq sample_7mer_TA16.fq

cutadapt -m 50 -g ^AAGTGTA -o ./trimmed_barcode/TA17.fq sample_7mer_TA17.fq

cutadapt -m 50 -g ^CCTGATA -o ./trimmed_barcode/TA18.fq sample_7mer_TA18.fq

cutadapt -m 50 -g ^TTCATTA -o ./trimmed_barcode/TA19.fq sample_7mer_TA19.fq

cutadapt -m 50 -g ^GGACGTA -o ./trimmed_barcode/TA20.fq sample_7mer_TA20.fq

cutadapt -m 50 -g ^ACATATA -o ./trimmed_barcode/TA21.fq sample_7mer_TA21.fq

cutadapt -m 50 -g ^CACGGTA -o ./trimmed_barcode/TA22.fq sample_7mer_TA22.fq

cutadapt -m 50 -g ^TGTCTTA -o ./trimmed_barcode/TA23.fq sample_7mer_TA23.fq

cutadapt -m 50 -g ^ATGAATA -o ./trimmed_barcode/TA24.fq sample_7mer_TA24.fq

cutadapt -m 50 -g ^GAGATTA -o ./trimmed_barcode/TA25.fq sample_7mer_TA25.fq

cutadapt -m 50 -g ^CTATGTA -o ./trimmed_barcode/TA26.fq sample_7mer_TA26.fq

cutadapt -m 50 -g ^ACTCGTA -o ./trimmed_barcode/TA27.fq sample_7mer_TA27.fq

cutadapt -m 50 -g ^GTCGATA -o ./trimmed_barcode/TA28.fq sample_7mer_TA28.fq

cutadapt -m 50 -g ^TAATTTA -o ./trimmed_barcode/TA29.fq sample_7mer_TA29.fq

cutadapt -m 50 -g ^CGCAATA -o ./trimmed_barcode/TA30.fq sample_7mer_TA30.fq

cutadapt -m 50 -g ^TCAGGTA -o ./trimmed_barcode/TA31.fq sample_7mer_TA31.fq

cutadapt -m 50 -g ^CTGGTTA -o ./trimmed_barcode/TA32.fq sample_7mer_TA32.fq

cutadapt -m 50 -g ^GATTATA -o ./trimmed_barcode/TA33.fq sample_7mer_TA33.fq

cutadapt -m 50 -g ^AGAATTA -o ./trimmed_barcode/TA34.fq sample_7mer_TA34.fq

cutadapt -m 50 -g ^TAGGATA -o ./trimmed_barcode/TA35.fq sample_7mer_TA35.fq

cutadapt -m 50 -g ^AGATATA -o ./trimmed_barcode/TA202.fq sample_7mer_TA202.fq

cutadapt -m 50 -g ^CCTTCTA -o ./trimmed_barcode/TA203.fq sample_7mer_TA203.fq

cutadapt -m 50 -g ^TGCTGTA -o ./trimmed_barcode/TA204.fq sample_7mer_TA204.fq

cutadapt -m 50 -g ^AACAATA -o ./trimmed_barcode/TA205.fq sample_7mer_TA205.fq

cutadapt -m 50 -g ^CCACGTA -o ./trimmed_barcode/TA206.fq sample_7mer_TA206.fq

cutadapt -m 50 -g ^TTGTCTA -o ./trimmed_barcode/TA207.fq sample_7mer_TA207.fq

cutadapt -m 50 -g ^GGTGATA -o ./trimmed_barcode/TA208.fq sample_7mer_TA208.fq

cutadapt -m 50 -g ^AATCCTA -o ./trimmed_barcode/TA209.fq sample_7mer_TA209.fq

cutadapt -m 50 -g ^CTCAGTA -o ./trimmed_barcode/TA210.fq sample_7mer_TA210.fq

cutadapt -m 50 -g ^TCAGATA -o ./trimmed_barcode/TA211.fq sample_7mer_TA211.fq

cutadapt -m 50 -g ^GAGTGTA -o ./trimmed_barcode/TA212.fq sample_7mer_TA212.fq

cutadapt -m 50 -g ^AGCGCTA -o ./trimmed_barcode/TA213.fq sample_7mer_TA213.fq

cutadapt -m 50 -g ^CCGAATA -o ./trimmed_barcode/TA214.fq sample_7mer_TA214.fq

cutadapt -m 50 -g ^TGACCTA -o ./trimmed_barcode/TA215.fq sample_7mer_TA215.fq

cutadapt -m 50 -g ^GTTCGTA -o ./trimmed_barcode/TA216.fq sample_7mer_TA216.fq

cutadapt -m 50 -g ^TATTATA -o ./trimmed_barcode/TA217.fq sample_7mer_TA217.fq

cutadapt -m 50 -g ^ACAACTA -o ./trimmed_barcode/TA218.fq sample_7mer_TA218.fq

cutadapt -m 50 -g ^GCCGGTA -o ./trimmed_barcode/TA219.fq sample_7mer_TA219.fq

cutadapt -m 50 -g ^GTCTATA -o ./trimmed_barcode/TA220.fq sample_7mer_TA220.fq

cutadapt -m 50 -g ^AGGAGTA -o ./trimmed_barcode/TA221.fq sample_7mer_TA221.fq

cutadapt -m 50 -g ^CAAGCTA -o ./trimmed_barcode/TA222.fq sample_7mer_TA222.fq

cutadapt -m 50 -g ^ATGCATA -o ./trimmed_barcode/TA223.fq sample_7mer_TA223.fq

cutadapt -m 50 -g ^TCTAGTA -o ./trimmed_barcode/TA224.fq sample_7mer_TA224.fq

cutadapt -m 50 -g ^CGCCATA -o ./trimmed_barcode/TA225.fq sample_7mer_TA225.fq

cutadapt -m 50 -g ^ATAGGTA -o ./trimmed_barcode/TA226.fq sample_7mer_TA226.fq

cutadapt -m 50 -g ^GCGCCTA -o ./trimmed_barcode/TA227.fq sample_7mer_TA227.fq

cutadapt -m 50 -g ^GAACATA -o ./trimmed_barcode/TA228.fq sample_7mer_TA228.fq

cutadapt -m 50 -g ^GCTCCACG -o ./trimmed_barcode/CG36.fq sample_8mer_CG36.fq

cutadapt -m 50 -g ^AGCCGTCG -o ./trimmed_barcode/CG37.fq sample_8mer_CG37.fq

cutadapt -m 50 -g ^CCTAGACG -o ./trimmed_barcode/CG38.fq sample_8mer_CG38.fq

cutadapt -m 50 -g ^ATGCCTCG -o ./trimmed_barcode/CG39.fq sample_8mer_CG39.fq

cutadapt -m 50 -g ^TGCTTACG -o ./trimmed_barcode/CG40.fq sample_8mer_CG40.fq

cutadapt -m 50 -g ^GAACATCG -o ./trimmed_barcode/CG41.fq sample_8mer_CG41.fq

cutadapt -m 50 -g ^CCGACTCG -o ./trimmed_barcode/CG42.fq sample_8mer_CG42.fq

cutadapt -m 50 -g ^TTCGGACG -o ./trimmed_barcode/CG43.fq sample_8mer_CG43.fq

cutadapt -m 50 -g ^AGTCTACG -o ./trimmed_barcode/CG44.fq sample_8mer_CG44.fq

cutadapt -m 50 -g ^GACTCTCG -o ./trimmed_barcode/CG45.fq sample_8mer_CG45.fq

cutadapt -m 50 -g ^CCATGTCG -o ./trimmed_barcode/CG46.fq sample_8mer_CG46.fq

cutadapt -m 50 -g ^CTTGTACG -o ./trimmed_barcode/CG47.fq sample_8mer_CG47.fq

cutadapt -m 50 -g ^TGCAATCG -o ./trimmed_barcode/CG48.fq sample_8mer_CG48.fq

cutadapt -m 50 -g ^GAGCTACG -o ./trimmed_barcode/CG49.fq sample_8mer_CG49.fq

cutadapt -m 50 -g ^ACAGATCG -o ./trimmed_barcode/CG50.fq sample_8mer_CG50.fq

cutadapt -m 50 -g ^TTGTCACG -o ./trimmed_barcode/CG51.fq sample_8mer_CG51.fq

cutadapt -m 50 -g ^AGTACTCG -o ./trimmed_barcode/CG52.fq sample_8mer_CG52.fq

cutadapt -m 50 -g ^GATCGGCG -o ./trimmed_barcode/CG53.fq sample_8mer_CG53.fq

cutadapt -m 50 -g ^CCGTAACG -o ./trimmed_barcode/CG54.fq sample_8mer_CG54.fq

cutadapt -m 50 -g ^GTCATACG -o ./trimmed_barcode/CG55.fq sample_8mer_CG55.fq

cutadapt -m 50 -g ^TGACGGCG -o ./trimmed_barcode/CG56.fq sample_8mer_CG56.fq

cutadapt -m 50 -g ^AACGTTCG -o ./trimmed_barcode/CG57.fq sample_8mer_CG57.fq

cutadapt -m 50 -g ^CTAACGCG -o ./trimmed_barcode/CG58.fq sample_8mer_CG58.fq

cutadapt -m 50 -g ^TCTGAACG -o ./trimmed_barcode/CG59.fq sample_8mer_CG59.fq

cutadapt -m 50 -g ^CAGCGTCG -o ./trimmed_barcode/CG60.fq sample_8mer_CG60.fq

cutadapt -m 50 -g ^GGATCACG -o ./trimmed_barcode/CG61.fq sample_8mer_CG61.fq

cutadapt -m 50 -g ^ACCATGCG -o ./trimmed_barcode/CG62.fq sample_8mer_CG62.fq

cutadapt -m 50 -g ^CTGGATCG -o ./trimmed_barcode/CG63.fq sample_8mer_CG63.fq

cutadapt -m 50 -g ^ACTTGGCG -o ./trimmed_barcode/CG64.fq sample_8mer_CG64.fq

cutadapt -m 50 -g ^TGAGCTCG -o ./trimmed_barcode/CG65.fq sample_8mer_CG65.fq

cutadapt -m 50 -g ^TGGCAACG -o ./trimmed_barcode/CG66.fq sample_8mer_CG66.fq

cutadapt -m 50 -g ^GTCTAGCG -o ./trimmed_barcode/CG67.fq sample_8mer_CG67.fq

cutadapt -m 50 -g ^GCTATTCG -o ./trimmed_barcode/CG68.fq sample_8mer_CG68.fq

cutadapt -m 50 -g ^CACTGACG -o ./trimmed_barcode/CG69.fq sample_8mer_CG69.fq

cutadapt -m 50 -g ^AATGCACG -o ./trimmed_barcode/CG70.fq sample_8mer_CG70.fq

cutadapt -m 50 -g ^GTACTGCG -o ./trimmed_barcode/CG71.fq sample_8mer_CG71.fq

cutadapt -m 50 -g ^TCGAGGCG -o ./trimmed_barcode/CG72.fq sample_8mer_CG72.fq

cutadapt -m 50 -g ^CGAATTCG -o ./trimmed_barcode/CG73.fq sample_8mer_CG73.fq

cutadapt -m 50 -g ^ATACGACG -o ./trimmed_barcode/CG74.fq sample_8mer_CG74.fq

cutadapt -m 50 -g ^CATTCGCG -o ./trimmed_barcode/CG229.fq sample_8mer_CG229.fq

cutadapt -m 50 -g ^TGATTCCG -o ./trimmed_barcode/CG230.fq sample_8mer_CG230.fq

cutadapt -m 50 -g ^CTCACGCG -o ./trimmed_barcode/CG231.fq sample_8mer_CG231.fq

cutadapt -m 50 -g ^AGTTGCCG -o ./trimmed_barcode/CG232.fq sample_8mer_CG232.fq

cutadapt -m 50 -g ^TAGGACCG -o ./trimmed_barcode/CG233.fq sample_8mer_CG233.fq

cutadapt -m 50 -g ^CCGATGCG -o ./trimmed_barcode/CG234.fq sample_8mer_CG234.fq

cutadapt -m 50 -g ^ATCGCCCG -o ./trimmed_barcode/CG235.fq sample_8mer_CG235.fq

cutadapt -m 50 -g ^CGATGGCG -o ./trimmed_barcode/CG236.fq sample_8mer_CG236.fq

cutadapt -m 50 -g ^TATAGCCG -o ./trimmed_barcode/CG237.fq sample_8mer_CG237.fq

cutadapt -m 50 -g ^GCTTAGCG -o ./trimmed_barcode/CG238.fq sample_8mer_CG238.fq

cutadapt -m 50 -g ^ATCCTGCG -o ./trimmed_barcode/CG239.fq sample_8mer_CG239.fq

cutadapt -m 50 -g ^TGGACCCG -o ./trimmed_barcode/CG240.fq sample_8mer_CG240.fq

cutadapt -m 50 -g ^GAAGTCCG -o ./trimmed_barcode/CG241.fq sample_8mer_CG241.fq

cutadapt -m 50 -g ^CTTCAGCG -o ./trimmed_barcode/CG242.fq sample_8mer_CG242.fq

cutadapt -m 50 -g ^ACCTCGCG -o ./trimmed_barcode/CG243.fq sample_8mer_CG243.fq

cutadapt -m 50 -g ^GTGAGCCG -o ./trimmed_barcode/CG244.fq sample_8mer_CG244.fq

cutadapt -m 50 -g ^TGAGCGCG -o ./trimmed_barcode/CG245.fq sample_8mer_CG245.fq

cutadapt -m 50 -g ^CACTGCCG -o ./trimmed_barcode/CG246.fq sample_8mer_CG246.fq

cutadapt -m 50 -g ^GAGCTGCG -o ./trimmed_barcode/CG248.fq sample_8mer_CG248.fq

cutadapt -m 50 -g ^AGCATCCG -o ./trimmed_barcode/CG249.fq sample_8mer_CG249.fq

cutadapt -m 50 -g ^CCTCGACG -o ./trimmed_barcode/CG250.fq sample_8mer_CG250.fq

cutadapt -m 50 -g ^GTATACCG -o ./trimmed_barcode/CG251.fq sample_8mer_CG251.fq

cutadapt -m 50 -g ^TTGGCACG -o ./trimmed_barcode/CG252.fq sample_8mer_CG252.fq

cutadapt -m 50 -g ^AGTACGCG -o ./trimmed_barcode/CG253.fq sample_8mer_CG253.fq

cutadapt -m 50 -g ^CACGTGCG -o ./trimmed_barcode/CG254.fq sample_8mer_CG254.fq

cutadapt -m 50 -g ^TCACGCCG -o ./trimmed_barcode/CG255.fq sample_8mer_CG255.fq

cutadapt -m 50 -g ^CGGTAACG -o ./trimmed_barcode/CG256.fq sample_8mer_CG256.fq

cutadapt -m 50 -g ^GAGTCCCG -o ./trimmed_barcode/CG257.fq sample_8mer_CG257.fq

cutadapt -m 50 -g ^ACAGTGCG -o ./trimmed_barcode/CG258.fq sample_8mer_CG258.fq

cutadapt -m 50 -g ^TTCCGACG -o ./trimmed_barcode/CG259.fq sample_8mer_CG259.fq

cutadapt -m 50 -g ^GGTAACCG -o ./trimmed_barcode/CG260.fq sample_8mer_CG260.fq

cutadapt -m 50 -g ^AATCGGCG -o ./trimmed_barcode/CG261.fq sample_8mer_CG261.fq

cutadapt -m 50 -g ^CTAGTACG -o ./trimmed_barcode/CG262.fq sample_8mer_CG262.fq

cutadapt -m 50 -g ^TCCAAGCG -o ./trimmed_barcode/CG263.fq sample_8mer_CG263.fq

cutadapt -m 50 -g ^GGCTCACG -o ./trimmed_barcode/CG264.fq sample_8mer_CG264.fq

cutadapt -m 50 -g ^ACGCTCCG -o ./trimmed_barcode/CG265.fq sample_8mer_CG265.fq

cutadapt -m 50 -g ^CGTGCACG -o ./trimmed_barcode/CG266.fq sample_8mer_CG266.fq

cutadapt -m 50 -g ^GTACGGCG -o ./trimmed_barcode/CG267.fq sample_8mer_CG267.fq

cutadapt -m 50 -g ^GCTCCATA -o ./trimmed_barcode/TA36.fq sample_8mer_TA36.fq

cutadapt -m 50 -g ^AGCCGTTA -o ./trimmed_barcode/TA37.fq sample_8mer_TA37.fq

cutadapt -m 50 -g ^CCTAGATA -o ./trimmed_barcode/TA38.fq sample_8mer_TA38.fq

cutadapt -m 50 -g ^ATGCCTTA -o ./trimmed_barcode/TA39.fq sample_8mer_TA39.fq

cutadapt -m 50 -g ^TGCTTATA -o ./trimmed_barcode/TA40.fq sample_8mer_TA40.fq

cutadapt -m 50 -g ^GAACATTA -o ./trimmed_barcode/TA41.fq sample_8mer_TA41.fq

cutadapt -m 50 -g ^CCGACTTA -o ./trimmed_barcode/TA42.fq sample_8mer_TA42.fq

cutadapt -m 50 -g ^TTCGGATA -o ./trimmed_barcode/TA43.fq sample_8mer_TA43.fq

cutadapt -m 50 -g ^AGTCTATA -o ./trimmed_barcode/TA44.fq sample_8mer_TA44.fq

cutadapt -m 50 -g ^GACTCTTA -o ./trimmed_barcode/TA45.fq sample_8mer_TA45.fq

cutadapt -m 50 -g ^CCATGTTA -o ./trimmed_barcode/TA46.fq sample_8mer_TA46.fq

cutadapt -m 50 -g ^CTTGTATA -o ./trimmed_barcode/TA47.fq sample_8mer_TA47.fq

cutadapt -m 50 -g ^TGCAATTA -o ./trimmed_barcode/TA48.fq sample_8mer_TA48.fq

cutadapt -m 50 -g ^GAGCTATA -o ./trimmed_barcode/TA49.fq sample_8mer_TA49.fq

cutadapt -m 50 -g ^ACAGATTA -o ./trimmed_barcode/TA50.fq sample_8mer_TA50.fq

cutadapt -m 50 -g ^TTGTCATA -o ./trimmed_barcode/TA51.fq sample_8mer_TA51.fq

cutadapt -m 50 -g ^AGTACTTA -o ./trimmed_barcode/TA52.fq sample_8mer_TA52.fq

cutadapt -m 50 -g ^GATCGGTA -o ./trimmed_barcode/TA53.fq sample_8mer_TA53.fq

cutadapt -m 50 -g ^CCGTAATA -o ./trimmed_barcode/TA54.fq sample_8mer_TA54.fq

cutadapt -m 50 -g ^GTCATATA -o ./trimmed_barcode/TA55.fq sample_8mer_TA55.fq

cutadapt -m 50 -g ^TGACGGTA -o ./trimmed_barcode/TA56.fq sample_8mer_TA56.fq

cutadapt -m 50 -g ^AACGTTTA -o ./trimmed_barcode/TA57.fq sample_8mer_TA57.fq

cutadapt -m 50 -g ^CTAACGTA -o ./trimmed_barcode/TA58.fq sample_8mer_TA58.fq

cutadapt -m 50 -g ^TCTGAATA -o ./trimmed_barcode/TA59.fq sample_8mer_TA59.fq

cutadapt -m 50 -g ^CAGCGTTA -o ./trimmed_barcode/TA60.fq sample_8mer_TA60.fq

cutadapt -m 50 -g ^GGATCATA -o ./trimmed_barcode/TA61.fq sample_8mer_TA61.fq

cutadapt -m 50 -g ^ACCATGTA -o ./trimmed_barcode/TA62.fq sample_8mer_TA62.fq

cutadapt -m 50 -g ^CTGGATTA -o ./trimmed_barcode/TA63.fq sample_8mer_TA63.fq

cutadapt -m 50 -g ^ACTTGGTA -o ./trimmed_barcode/TA64.fq sample_8mer_TA64.fq

cutadapt -m 50 -g ^TGAGCTTA -o ./trimmed_barcode/TA65.fq sample_8mer_TA65.fq

cutadapt -m 50 -g ^TGGCAATA -o ./trimmed_barcode/TA66.fq sample_8mer_TA66.fq

cutadapt -m 50 -g ^GTCTAGTA -o ./trimmed_barcode/TA67.fq sample_8mer_TA67.fq

cutadapt -m 50 -g ^GCTATTTA -o ./trimmed_barcode/TA68.fq sample_8mer_TA68.fq

cutadapt -m 50 -g ^CACTGATA -o ./trimmed_barcode/TA69.fq sample_8mer_TA69.fq

cutadapt -m 50 -g ^AATGCATA -o ./trimmed_barcode/TA70.fq sample_8mer_TA70.fq

cutadapt -m 50 -g ^GTACTGTA -o ./trimmed_barcode/TA71.fq sample_8mer_TA71.fq

cutadapt -m 50 -g ^TCGAGGTA -o ./trimmed_barcode/TA72.fq sample_8mer_TA72.fq

cutadapt -m 50 -g ^CGAATTTA -o ./trimmed_barcode/TA73.fq sample_8mer_TA73.fq

cutadapt -m 50 -g ^ATACGATA -o ./trimmed_barcode/TA74.fq sample_8mer_TA74.fq

cutadapt -m 50 -g ^CATTCGTA -o ./trimmed_barcode/TA229.fq sample_8mer_TA229.fq

cutadapt -m 50 -g ^TGATTCTA -o ./trimmed_barcode/TA230.fq sample_8mer_TA230.fq

cutadapt -m 50 -g ^CTCACGTA -o ./trimmed_barcode/TA231.fq sample_8mer_TA231.fq

cutadapt -m 50 -g ^AGTTGCTA -o ./trimmed_barcode/TA232.fq sample_8mer_TA232.fq

cutadapt -m 50 -g ^TAGGACTA -o ./trimmed_barcode/TA233.fq sample_8mer_TA233.fq

cutadapt -m 50 -g ^CCGATGTA -o ./trimmed_barcode/TA234.fq sample_8mer_TA234.fq

cutadapt -m 50 -g ^ATCGCCTA -o ./trimmed_barcode/TA235.fq sample_8mer_TA235.fq

cutadapt -m 50 -g ^CGATGGTA -o ./trimmed_barcode/TA236.fq sample_8mer_TA236.fq

cutadapt -m 50 -g ^TATAGCTA -o ./trimmed_barcode/TA237.fq sample_8mer_TA237.fq

cutadapt -m 50 -g ^GCTTAGTA -o ./trimmed_barcode/TA238.fq sample_8mer_TA238.fq

cutadapt -m 50 -g ^ATCCTGTA -o ./trimmed_barcode/TA239.fq sample_8mer_TA239.fq

cutadapt -m 50 -g ^TGGACCTA -o ./trimmed_barcode/TA240.fq sample_8mer_TA240.fq

cutadapt -m 50 -g ^GAAGTCTA -o ./trimmed_barcode/TA241.fq sample_8mer_TA241.fq

cutadapt -m 50 -g ^CTTCAGTA -o ./trimmed_barcode/TA242.fq sample_8mer_TA242.fq

cutadapt -m 50 -g ^ACCTCGTA -o ./trimmed_barcode/TA243.fq sample_8mer_TA243.fq

cutadapt -m 50 -g ^GTGAGCTA -o ./trimmed_barcode/TA244.fq sample_8mer_TA244.fq

cutadapt -m 50 -g ^TGAGCGTA -o ./trimmed_barcode/TA245.fq sample_8mer_TA245.fq

cutadapt -m 50 -g ^CACTGCTA -o ./trimmed_barcode/TA246.fq sample_8mer_TA246.fq

cutadapt -m 50 -g ^GAGCTGTA -o ./trimmed_barcode/TA248.fq sample_8mer_TA248.fq

cutadapt -m 50 -g ^AGCATCTA -o ./trimmed_barcode/TA249.fq sample_8mer_TA249.fq

cutadapt -m 50 -g ^CCTCGATA -o ./trimmed_barcode/TA250.fq sample_8mer_TA250.fq

cutadapt -m 50 -g ^GTATACTA -o ./trimmed_barcode/TA251.fq sample_8mer_TA251.fq

cutadapt -m 50 -g ^TTGGCATA -o ./trimmed_barcode/TA252.fq sample_8mer_TA252.fq

cutadapt -m 50 -g ^AGTACGTA -o ./trimmed_barcode/TA253.fq sample_8mer_TA253.fq

cutadapt -m 50 -g ^CACGTGTA -o ./trimmed_barcode/TA254.fq sample_8mer_TA254.fq

cutadapt -m 50 -g ^TCACGCTA -o ./trimmed_barcode/TA255.fq sample_8mer_TA255.fq

cutadapt -m 50 -g ^CGGTAATA -o ./trimmed_barcode/TA256.fq sample_8mer_TA256.fq

cutadapt -m 50 -g ^GAGTCCTA -o ./trimmed_barcode/TA257.fq sample_8mer_TA257.fq

cutadapt -m 50 -g ^ACAGTGTA -o ./trimmed_barcode/TA258.fq sample_8mer_TA258.fq

cutadapt -m 50 -g ^TTCCGATA -o ./trimmed_barcode/TA259.fq sample_8mer_TA259.fq

cutadapt -m 50 -g ^GGTAACTA -o ./trimmed_barcode/TA260.fq sample_8mer_TA260.fq

cutadapt -m 50 -g ^AATCGGTA -o ./trimmed_barcode/TA261.fq sample_8mer_TA261.fq

cutadapt -m 50 -g ^CTAGTATA -o ./trimmed_barcode/TA262.fq sample_8mer_TA262.fq

cutadapt -m 50 -g ^TCCAAGTA -o ./trimmed_barcode/TA263.fq sample_8mer_TA263.fq

cutadapt -m 50 -g ^GGCTCATA -o ./trimmed_barcode/TA264.fq sample_8mer_TA264.fq

cutadapt -m 50 -g ^ACGCTCTA -o ./trimmed_barcode/TA265.fq sample_8mer_TA265.fq

cutadapt -m 50 -g ^CGTGCATA -o ./trimmed_barcode/TA266.fq sample_8mer_TA266.fq

cutadapt -m 50 -g ^GTACGGTA -o ./trimmed_barcode/TA267.fq sample_8mer_TA267.fq

cutadapt -m 50 -g ^TACGATACG -o ./trimmed_barcode/CG75.fq sample_9mer_CG75.fq

cutadapt -m 50 -g ^AGTTCTTCG -o ./trimmed_barcode/CG76.fq sample_9mer_CG76.fq

cutadapt -m 50 -g ^GAGACATCG -o ./trimmed_barcode/CG77.fq sample_9mer_CG77.fq

cutadapt -m 50 -g ^CCTCAGACG -o ./trimmed_barcode/CG78.fq sample_9mer_CG78.fq

cutadapt -m 50 -g ^TTCTGATCG -o ./trimmed_barcode/CG79.fq sample_9mer_CG79.fq

cutadapt -m 50 -g ^GGCGTTACG -o ./trimmed_barcode/CG80.fq sample_9mer_CG80.fq

cutadapt -m 50 -g ^AAGCAGTCG -o ./trimmed_barcode/CG81.fq sample_9mer_CG81.fq

cutadapt -m 50 -g ^CCAATGACG -o ./trimmed_barcode/CG82.fq sample_9mer_CG82.fq

cutadapt -m 50 -g ^TTGGCATCG -o ./trimmed_barcode/CG83.fq sample_9mer_CG83.fq

cutadapt -m 50 -g ^CGTTGTACG -o ./trimmed_barcode/CG84.fq sample_9mer_CG84.fq

cutadapt -m 50 -g ^GAACAATCG -o ./trimmed_barcode/CG85.fq sample_9mer_CG85.fq

cutadapt -m 50 -g ^ACCTCGACG -o ./trimmed_barcode/CG86.fq sample_9mer_CG86.fq

cutadapt -m 50 -g ^CTTAGTTCG -o ./trimmed_barcode/CG87.fq sample_9mer_CG87.fq

cutadapt -m 50 -g ^TGGCTAACG -o ./trimmed_barcode/CG88.fq sample_9mer_CG88.fq

cutadapt -m 50 -g ^GAAGCTACG -o ./trimmed_barcode/CG89.fq sample_9mer_CG89.fq

cutadapt -m 50 -g ^ACCAAGTCG -o ./trimmed_barcode/CG90.fq sample_9mer_CG90.fq

cutadapt -m 50 -g ^CTTGTTACG -o ./trimmed_barcode/CG91.fq sample_9mer_CG91.fq

cutadapt -m 50 -g ^GCGTGATCG -o ./trimmed_barcode/CG92.fq sample_9mer_CG92.fq

cutadapt -m 50 -g ^TGAACGACG -o ./trimmed_barcode/CG93.fq sample_9mer_CG93.fq

cutadapt -m 50 -g ^AACCGATCG -o ./trimmed_barcode/CG94.fq sample_9mer_CG94.fq

cutadapt -m 50 -g ^CTGTAGACG -o ./trimmed_barcode/CG95.fq sample_9mer_CG95.fq

cutadapt -m 50 -g ^TATGCTTCG -o ./trimmed_barcode/CG96.fq sample_9mer_CG96.fq

cutadapt -m 50 -g ^GGACTGACG -o ./trimmed_barcode/CG97.fq sample_9mer_CG97.fq

cutadapt -m 50 -g ^ACCTGTTCG -o ./trimmed_barcode/CG98.fq sample_9mer_CG98.fq

cutadapt -m 50 -g ^CTGATAACG -o ./trimmed_barcode/CG99.fq sample_9mer_CG99.fq

cutadapt -m 50 -g ^TCTGAGTCG -o ./trimmed_barcode/CG100.fq sample_9mer_CG100.fq

cutadapt -m 50 -g ^GGACCTTCG -o ./trimmed_barcode/CG101.fq sample_9mer_CG101.fq

cutadapt -m 50 -g ^AACGTAACG -o ./trimmed_barcode/CG102.fq sample_9mer_CG102.fq

cutadapt -m 50 -g ^CGTAAGTCG -o ./trimmed_barcode/CG103.fq sample_9mer_CG103.fq

cutadapt -m 50 -g ^TCGTGTACG -o ./trimmed_barcode/CG104.fq sample_9mer_CG104.fq

cutadapt -m 50 -g ^GTAACAACG -o ./trimmed_barcode/CG105.fq sample_9mer_CG105.fq

cutadapt -m 50 -g ^ATCCTGTCG -o ./trimmed_barcode/CG106.fq sample_9mer_CG106.fq

cutadapt -m 50 -g ^CATGGAACG -o ./trimmed_barcode/CG107.fq sample_9mer_CG107.fq

cutadapt -m 50 -g ^TGGCATTCG -o ./trimmed_barcode/CG108.fq sample_9mer_CG108.fq

cutadapt -m 50 -g ^GAATACACG -o ./trimmed_barcode/CG109.fq sample_9mer_CG109.fq

cutadapt -m 50 -g ^ACTAGATCG -o ./trimmed_barcode/CG110.fq sample_9mer_CG110.fq

cutadapt -m 50 -g ^TTCGCGACG -o ./trimmed_barcode/CG111.fq sample_9mer_CG111.fq

cutadapt -m 50 -g ^AGGTTCTCG -o ./trimmed_barcode/CG112.fq sample_9mer_CG112.fq

cutadapt -m 50 -g ^GACAATTCG -o ./trimmed_barcode/CG113.fq sample_9mer_CG113.fq

cutadapt -m 50 -g ^TAGTACCCG -o ./trimmed_barcode/CG268.fq sample_9mer_CG268.fq

cutadapt -m 50 -g ^AACATCGCG -o ./trimmed_barcode/CG269.fq sample_9mer_CG269.fq

cutadapt -m 50 -g ^CCATAGCCG -o ./trimmed_barcode/CG270.fq sample_9mer_CG270.fq

cutadapt -m 50 -g ^TTGACAGCG -o ./trimmed_barcode/CG271.fq sample_9mer_CG271.fq

cutadapt -m 50 -g ^AGTGGCCCG -o ./trimmed_barcode/CG272.fq sample_9mer_CG272.fq

cutadapt -m 50 -g ^GACTAGGCG -o ./trimmed_barcode/CG273.fq sample_9mer_CG273.fq

cutadapt -m 50 -g ^CCAATAGCG -o ./trimmed_barcode/CG274.fq sample_9mer_CG274.fq

cutadapt -m 50 -g ^ATTCCGCCG -o ./trimmed_barcode/CG275.fq sample_9mer_CG275.fq

cutadapt -m 50 -g ^TGGAGCCCG -o ./trimmed_barcode/CG276.fq sample_9mer_CG276.fq

cutadapt -m 50 -g ^GACGTAGCG -o ./trimmed_barcode/CG277.fq sample_9mer_CG277.fq

cutadapt -m 50 -g ^CCATCCGCG -o ./trimmed_barcode/CG278.fq sample_9mer_CG278.fq

cutadapt -m 50 -g ^TTGGAGCCG -o ./trimmed_barcode/CG279.fq sample_9mer_CG279.fq

cutadapt -m 50 -g ^GGTCGACCG -o ./trimmed_barcode/CG280.fq sample_9mer_CG280.fq

cutadapt -m 50 -g ^AATACGGCG -o ./trimmed_barcode/CG281.fq sample_9mer_CG281.fq

cutadapt -m 50 -g ^CCAGTCCCG -o ./trimmed_barcode/CG282.fq sample_9mer_CG282.fq

cutadapt -m 50 -g ^TTCCAGGCG -o ./trimmed_barcode/CG283.fq sample_9mer_CG283.fq

cutadapt -m 50 -g ^AGGTGCGCG -o ./trimmed_barcode/CG284.fq sample_9mer_CG284.fq

cutadapt -m 50 -g ^GAACTACCG -o ./trimmed_barcode/CG285.fq sample_9mer_CG285.fq

cutadapt -m 50 -g ^CCTTGACCG -o ./trimmed_barcode/CG286.fq sample_9mer_CG286.fq

cutadapt -m 50 -g ^TGCACCGCG -o ./trimmed_barcode/CG287.fq sample_9mer_CG287.fq

cutadapt -m 50 -g ^GTGGACGCG -o ./trimmed_barcode/CG288.fq sample_9mer_CG288.fq

cutadapt -m 50 -g ^AACCTGCCG -o ./trimmed_barcode/CG289.fq sample_9mer_CG289.fq

cutadapt -m 50 -g ^CCTGAAGCG -o ./trimmed_barcode/CG290.fq sample_9mer_CG290.fq

cutadapt -m 50 -g ^TTATCGCCG -o ./trimmed_barcode/CG291.fq sample_9mer_CG291.fq

cutadapt -m 50 -g ^GTGAGACCG -o ./trimmed_barcode/CG292.fq sample_9mer_CG292.fq

cutadapt -m 50 -g ^AGACTCGCG -o ./trimmed_barcode/CG293.fq sample_9mer_CG293.fq

cutadapt -m 50 -g ^CACTCGCCG -o ./trimmed_barcode/CG294.fq sample_9mer_CG294.fq

cutadapt -m 50 -g ^TCTAAGGCG -o ./trimmed_barcode/CG295.fq sample_9mer_CG295.fq

cutadapt -m 50 -g ^TGAGGACCG -o ./trimmed_barcode/CG296.fq sample_9mer_CG296.fq

cutadapt -m 50 -g ^GAGCTCGCG -o ./trimmed_barcode/CG297.fq sample_9mer_CG297.fq

cutadapt -m 50 -g ^ACCTGAGCG -o ./trimmed_barcode/CG298.fq sample_9mer_CG298.fq

cutadapt -m 50 -g ^CTGAACCCG -o ./trimmed_barcode/CG299.fq sample_9mer_CG299.fq

cutadapt -m 50 -g ^GATGCGCCG -o ./trimmed_barcode/CG300.fq sample_9mer_CG300.fq

cutadapt -m 50 -g ^AGAACTGCG -o ./trimmed_barcode/CG301.fq sample_9mer_CG301.fq

cutadapt -m 50 -g ^CCGCTACCG -o ./trimmed_barcode/CG302.fq sample_9mer_CG302.fq

cutadapt -m 50 -g ^TGTGACGCG -o ./trimmed_barcode/CG303.fq sample_9mer_CG303.fq

cutadapt -m 50 -g ^ATGTGGCCG -o ./trimmed_barcode/CG304.fq sample_9mer_CG304.fq

cutadapt -m 50 -g ^GACACTGCG -o ./trimmed_barcode/CG305.fq sample_9mer_CG305.fq

cutadapt -m 50 -g ^CTACAAGCG -o ./trimmed_barcode/CG306.fq sample_9mer_CG306.fq

cutadapt -m 50 -g ^TACGATATA -o ./trimmed_barcode/TA75.fq sample_9mer_TA75.fq

cutadapt -m 50 -g ^AGTTCTTTA -o ./trimmed_barcode/TA76.fq sample_9mer_TA76.fq

cutadapt -m 50 -g ^GAGACATTA -o ./trimmed_barcode/TA77.fq sample_9mer_TA77.fq

cutadapt -m 50 -g ^CCTCAGATA -o ./trimmed_barcode/TA78.fq sample_9mer_TA78.fq

cutadapt -m 50 -g ^TTCTGATTA -o ./trimmed_barcode/TA79.fq sample_9mer_TA79.fq

cutadapt -m 50 -g ^GGCGTTATA -o ./trimmed_barcode/TA80.fq sample_9mer_TA80.fq

cutadapt -m 50 -g ^AAGCAGTTA -o ./trimmed_barcode/TA81.fq sample_9mer_TA81.fq

cutadapt -m 50 -g ^CCAATGATA -o ./trimmed_barcode/TA82.fq sample_9mer_TA82.fq

cutadapt -m 50 -g ^TTGGCATTA -o ./trimmed_barcode/TA83.fq sample_9mer_TA83.fq

cutadapt -m 50 -g ^CGTTGTATA -o ./trimmed_barcode/TA84.fq sample_9mer_TA84.fq

cutadapt -m 50 -g ^GAACAATTA -o ./trimmed_barcode/TA85.fq sample_9mer_TA85.fq

cutadapt -m 50 -g ^ACCTCGATA -o ./trimmed_barcode/TA86.fq sample_9mer_TA86.fq

cutadapt -m 50 -g ^CTTAGTTTA -o ./trimmed_barcode/TA87.fq sample_9mer_TA87.fq

cutadapt -m 50 -g ^TGGCTAATA -o ./trimmed_barcode/TA88.fq sample_9mer_TA88.fq

cutadapt -m 50 -g ^GAAGCTATA -o ./trimmed_barcode/TA89.fq sample_9mer_TA89.fq

cutadapt -m 50 -g ^ACCAAGTTA -o ./trimmed_barcode/TA90.fq sample_9mer_TA90.fq

cutadapt -m 50 -g ^CTTGTTATA -o ./trimmed_barcode/TA91.fq sample_9mer_TA91.fq

cutadapt -m 50 -g ^GCGTGATTA -o ./trimmed_barcode/TA92.fq sample_9mer_TA92.fq

cutadapt -m 50 -g ^TGAACGATA -o ./trimmed_barcode/TA93.fq sample_9mer_TA93.fq

cutadapt -m 50 -g ^AACCGATTA -o ./trimmed_barcode/TA94.fq sample_9mer_TA94.fq

cutadapt -m 50 -g ^CTGTAGATA -o ./trimmed_barcode/TA95.fq sample_9mer_TA95.fq

cutadapt -m 50 -g ^TATGCTTTA -o ./trimmed_barcode/TA96.fq sample_9mer_TA96.fq

cutadapt -m 50 -g ^GGACTGATA -o ./trimmed_barcode/TA97.fq sample_9mer_TA97.fq

cutadapt -m 50 -g ^ACCTGTTTA -o ./trimmed_barcode/TA98.fq sample_9mer_TA98.fq

cutadapt -m 50 -g ^CTGATAATA -o ./trimmed_barcode/TA99.fq sample_9mer_TA99.fq

cutadapt -m 50 -g ^TCTGAGTTA -o ./trimmed_barcode/TA100.fq sample_9mer_TA100.fq

cutadapt -m 50 -g ^GGACCTTTA -o ./trimmed_barcode/TA101.fq sample_9mer_TA101.fq

cutadapt -m 50 -g ^AACGTAATA -o ./trimmed_barcode/TA102.fq sample_9mer_TA102.fq

cutadapt -m 50 -g ^CGTAAGTTA -o ./trimmed_barcode/TA103.fq sample_9mer_TA103.fq

cutadapt -m 50 -g ^TCGTGTATA -o ./trimmed_barcode/TA104.fq sample_9mer_TA104.fq

cutadapt -m 50 -g ^GTAACAATA -o ./trimmed_barcode/TA105.fq sample_9mer_TA105.fq

cutadapt -m 50 -g ^ATCCTGTTA -o ./trimmed_barcode/TA106.fq sample_9mer_TA106.fq

cutadapt -m 50 -g ^CATGGAATA -o ./trimmed_barcode/TA107.fq sample_9mer_TA107.fq

cutadapt -m 50 -g ^TGGCATTTA -o ./trimmed_barcode/TA108.fq sample_9mer_TA108.fq

cutadapt -m 50 -g ^GAATACATA -o ./trimmed_barcode/TA109.fq sample_9mer_TA109.fq

cutadapt -m 50 -g ^ACTAGATTA -o ./trimmed_barcode/TA110.fq sample_9mer_TA110.fq

cutadapt -m 50 -g ^TTCGCGATA -o ./trimmed_barcode/TA111.fq sample_9mer_TA111.fq

cutadapt -m 50 -g ^AGGTTCTTA -o ./trimmed_barcode/TA112.fq sample_9mer_TA112.fq

cutadapt -m 50 -g ^GACAATTTA -o ./trimmed_barcode/TA113.fq sample_9mer_TA113.fq

cutadapt -m 50 -g ^TAGTACCTA -o ./trimmed_barcode/TA268.fq sample_9mer_TA268.fq

cutadapt -m 50 -g ^AACATCGTA -o ./trimmed_barcode/TA269.fq sample_9mer_TA269.fq

cutadapt -m 50 -g ^CCATAGCTA -o ./trimmed_barcode/TA270.fq sample_9mer_TA270.fq

cutadapt -m 50 -g ^TTGACAGTA -o ./trimmed_barcode/TA271.fq sample_9mer_TA271.fq

cutadapt -m 50 -g ^AGTGGCCTA -o ./trimmed_barcode/TA272.fq sample_9mer_TA272.fq

cutadapt -m 50 -g ^GACTAGGTA -o ./trimmed_barcode/TA273.fq sample_9mer_TA273.fq

cutadapt -m 50 -g ^CCAATAGTA -o ./trimmed_barcode/TA274.fq sample_9mer_TA274.fq

cutadapt -m 50 -g ^ATTCCGCTA -o ./trimmed_barcode/TA275.fq sample_9mer_TA275.fq

cutadapt -m 50 -g ^TGGAGCCTA -o ./trimmed_barcode/TA276.fq sample_9mer_TA276.fq

cutadapt -m 50 -g ^GACGTAGTA -o ./trimmed_barcode/TA277.fq sample_9mer_TA277.fq

cutadapt -m 50 -g ^CCATCCGTA -o ./trimmed_barcode/TA278.fq sample_9mer_TA278.fq

cutadapt -m 50 -g ^TTGGAGCTA -o ./trimmed_barcode/TA279.fq sample_9mer_TA279.fq

cutadapt -m 50 -g ^GGTCGACTA -o ./trimmed_barcode/TA280.fq sample_9mer_TA280.fq

cutadapt -m 50 -g ^AATACGGTA -o ./trimmed_barcode/TA281.fq sample_9mer_TA281.fq

cutadapt -m 50 -g ^CCAGTCCTA -o ./trimmed_barcode/TA282.fq sample_9mer_TA282.fq

cutadapt -m 50 -g ^TTCCAGGTA -o ./trimmed_barcode/TA283.fq sample_9mer_TA283.fq

cutadapt -m 50 -g ^AGGTGCGTA -o ./trimmed_barcode/TA284.fq sample_9mer_TA284.fq

cutadapt -m 50 -g ^GAACTACTA -o ./trimmed_barcode/TA285.fq sample_9mer_TA285.fq

cutadapt -m 50 -g ^CCTTGACTA -o ./trimmed_barcode/TA286.fq sample_9mer_TA286.fq

cutadapt -m 50 -g ^TGCACCGTA -o ./trimmed_barcode/TA287.fq sample_9mer_TA287.fq

cutadapt -m 50 -g ^GTGGACGTA -o ./trimmed_barcode/TA288.fq sample_9mer_TA288.fq

cutadapt -m 50 -g ^CCACCGATCG -o ./trimmed_barcode/CG114.fq sample_10mer_CG114.fq

cutadapt -m 50 -g ^CTTGGATACG -o ./trimmed_barcode/CG115.fq sample_10mer_CG115.fq

cutadapt -m 50 -g ^TGGTCTAACG -o ./trimmed_barcode/CG116.fq sample_10mer_CG116.fq

cutadapt -m 50 -g ^GAATTCTTCG -o ./trimmed_barcode/CG117.fq sample_10mer_CG117.fq

cutadapt -m 50 -g ^ACCAAGATCG -o ./trimmed_barcode/CG118.fq sample_10mer_CG118.fq

cutadapt -m 50 -g ^TTGCTATACG -o ./trimmed_barcode/CG119.fq sample_10mer_CG119.fq

cutadapt -m 50 -g ^GGTCGTAACG -o ./trimmed_barcode/CG120.fq sample_10mer_CG120.fq

cutadapt -m 50 -g ^AACGACATCG -o ./trimmed_barcode/CG121.fq sample_10mer_CG121.fq

cutadapt -m 50 -g ^CCAACGTACG -o ./trimmed_barcode/CG122.fq sample_10mer_CG122.fq

cutadapt -m 50 -g ^CTGTTAATCG -o ./trimmed_barcode/CG123.fq sample_10mer_CG123.fq

cutadapt -m 50 -g ^TGTGGCTACG -o ./trimmed_barcode/CG124.fq sample_10mer_CG124.fq

cutadapt -m 50 -g ^GAACCTATCG -o ./trimmed_barcode/CG125.fq sample_10mer_CG125.fq

cutadapt -m 50 -g ^ACCTAGTACG -o ./trimmed_barcode/CG126.fq sample_10mer_CG126.fq

cutadapt -m 50 -g ^CGTAGATTCG -o ./trimmed_barcode/CG127.fq sample_10mer_CG127.fq

cutadapt -m 50 -g ^TTCGTTAACG -o ./trimmed_barcode/CG128.fq sample_10mer_CG128.fq

cutadapt -m 50 -g ^GAGAACGTCG -o ./trimmed_barcode/CG129.fq sample_10mer_CG129.fq

cutadapt -m 50 -g ^ACACTGTACG -o ./trimmed_barcode/CG130.fq sample_10mer_CG130.fq

cutadapt -m 50 -g ^TTGGCTATCG -o ./trimmed_barcode/CG131.fq sample_10mer_CG131.fq

cutadapt -m 50 -g ^GGCTGAATCG -o ./trimmed_barcode/CG132.fq sample_10mer_CG132.fq

cutadapt -m 50 -g ^AATAACGACG -o ./trimmed_barcode/CG133.fq sample_10mer_CG133.fq

cutadapt -m 50 -g ^CCACGATACG -o ./trimmed_barcode/CG134.fq sample_10mer_CG134.fq

cutadapt -m 50 -g ^TGTTCGATCG -o ./trimmed_barcode/CG135.fq sample_10mer_CG135.fq

cutadapt -m 50 -g ^GTGGCTGACG -o ./trimmed_barcode/CG136.fq sample_10mer_CG136.fq

cutadapt -m 50 -g ^AACATGTTCG -o ./trimmed_barcode/CG137.fq sample_10mer_CG137.fq

cutadapt -m 50 -g ^CCACAAGTCG -o ./trimmed_barcode/CG138.fq sample_10mer_CG138.fq

cutadapt -m 50 -g ^TTGTTCAACG -o ./trimmed_barcode/CG139.fq sample_10mer_CG139.fq

cutadapt -m 50 -g ^CGAGGTTACG -o ./trimmed_barcode/CG140.fq sample_10mer_CG140.fq

cutadapt -m 50 -g ^GATACAATCG -o ./trimmed_barcode/CG141.fq sample_10mer_CG141.fq

cutadapt -m 50 -g ^ACCTACGTCG -o ./trimmed_barcode/CG142.fq sample_10mer_CG142.fq

cutadapt -m 50 -g ^GTTCTGTACG -o ./trimmed_barcode/CG143.fq sample_10mer_CG143.fq

cutadapt -m 50 -g ^TGGCGTGACG -o ./trimmed_barcode/CG144.fq sample_10mer_CG144.fq

cutadapt -m 50 -g ^AACGCATTCG -o ./trimmed_barcode/CG145.fq sample_10mer_CG145.fq

cutadapt -m 50 -g ^CCAAGCATCG -o ./trimmed_barcode/CG146.fq sample_10mer_CG146.fq

cutadapt -m 50 -g ^CTTGTGAACG -o ./trimmed_barcode/CG147.fq sample_10mer_CG147.fq

cutadapt -m 50 -g ^AGGCATTACG -o ./trimmed_barcode/CG148.fq sample_10mer_CG148.fq

cutadapt -m 50 -g ^TAATACGTCG -o ./trimmed_barcode/CG149.fq sample_10mer_CG149.fq

cutadapt -m 50 -g ^GCCACATTCG -o ./trimmed_barcode/CG150.fq sample_10mer_CG150.fq

cutadapt -m 50 -g ^CTGTTGGACG -o ./trimmed_barcode/CG151.fq sample_10mer_CG151.fq

cutadapt -m 50 -g ^GGTGCTATCG -o ./trimmed_barcode/CG152.fq sample_10mer_CG152.fq

cutadapt -m 50 -g ^ACCTTGCCCG -o ./trimmed_barcode/CG307.fq sample_10mer_CG307.fq

cutadapt -m 50 -g ^TATGGCCGCG -o ./trimmed_barcode/CG308.fq sample_10mer_CG308.fq

cutadapt -m 50 -g ^GGAACTGCCG -o ./trimmed_barcode/CG309.fq sample_10mer_CG309.fq

cutadapt -m 50 -g ^CCTCAAGGCG -o ./trimmed_barcode/CG310.fq sample_10mer_CG310.fq

cutadapt -m 50 -g ^ATCTTCCGCG -o ./trimmed_barcode/CG311.fq sample_10mer_CG311.fq

cutadapt -m 50 -g ^TAGCGGCCCG -o ./trimmed_barcode/CG312.fq sample_10mer_CG312.fq

cutadapt -m 50 -g ^GGAGTAGCCG -o ./trimmed_barcode/CG313.fq sample_10mer_CG313.fq

cutadapt -m 50 -g ^CCTTATCGCG -o ./trimmed_barcode/CG314.fq sample_10mer_CG314.fq

cutadapt -m 50 -g ^TTCACCGCCG -o ./trimmed_barcode/CG315.fq sample_10mer_CG315.fq

cutadapt -m 50 -g ^AGGTGGCGCG -o ./trimmed_barcode/CG316.fq sample_10mer_CG316.fq

cutadapt -m 50 -g ^GAAGATGCCG -o ./trimmed_barcode/CG317.fq sample_10mer_CG317.fq

cutadapt -m 50 -g ^CCTGCACGCG -o ./trimmed_barcode/CG318.fq sample_10mer_CG318.fq

cutadapt -m 50 -g ^TTGATGGCCG -o ./trimmed_barcode/CG319.fq sample_10mer_CG319.fq

cutadapt -m 50 -g ^ATCCGCGGCG -o ./trimmed_barcode/CG320.fq sample_10mer_CG320.fq

cutadapt -m 50 -g ^GGAATACGCG -o ./trimmed_barcode/CG321.fq sample_10mer_CG321.fq

cutadapt -m 50 -g ^CACCATGCCG -o ./trimmed_barcode/CG322.fq sample_10mer_CG322.fq

cutadapt -m 50 -g ^ACTTCGCGCG -o ./trimmed_barcode/CG323.fq sample_10mer_CG323.fq

cutadapt -m 50 -g ^TACGGCGCCG -o ./trimmed_barcode/CG324.fq sample_10mer_CG324.fq

cutadapt -m 50 -g ^CGGAATCGCG -o ./trimmed_barcode/CG325.fq sample_10mer_CG325.fq

cutadapt -m 50 -g ^GCACTACCCG -o ./trimmed_barcode/CG326.fq sample_10mer_CG326.fq

cutadapt -m 50 -g ^ATTGCCGCCG -o ./trimmed_barcode/CG327.fq sample_10mer_CG327.fq

cutadapt -m 50 -g ^TCATGGCGCG -o ./trimmed_barcode/CG328.fq sample_10mer_CG328.fq

cutadapt -m 50 -g ^CGGATAGCCG -o ./trimmed_barcode/CG329.fq sample_10mer_CG329.fq

cutadapt -m 50 -g ^GACCATCGCG -o ./trimmed_barcode/CG330.fq sample_10mer_CG330.fq

cutadapt -m 50 -g ^ATGTCCGGCG -o ./trimmed_barcode/CG331.fq sample_10mer_CG331.fq

cutadapt -m 50 -g ^TGTGGAGCCG -o ./trimmed_barcode/CG332.fq sample_10mer_CG332.fq

cutadapt -m 50 -g ^GAACTGCGCG -o ./trimmed_barcode/CG333.fq sample_10mer_CG333.fq

cutadapt -m 50 -g ^CCTGACGCCG -o ./trimmed_barcode/CG334.fq sample_10mer_CG334.fq

cutadapt -m 50 -g ^TTCACTCGCG -o ./trimmed_barcode/CG335.fq sample_10mer_CG335.fq

cutadapt -m 50 -g ^GGATGGCCCG -o ./trimmed_barcode/CG336.fq sample_10mer_CG336.fq

cutadapt -m 50 -g ^AAGAATGCCG -o ./trimmed_barcode/CG337.fq sample_10mer_CG337.fq

cutadapt -m 50 -g ^CCGTTAGGCG -o ./trimmed_barcode/CG338.fq sample_10mer_CG338.fq

cutadapt -m 50 -g ^ATCCGGCCCG -o ./trimmed_barcode/CG339.fq sample_10mer_CG339.fq

cutadapt -m 50 -g ^TGTGCCAGCG -o ./trimmed_barcode/CG340.fq sample_10mer_CG340.fq

cutadapt -m 50 -g ^GAACCTCCCG -o ./trimmed_barcode/CG341.fq sample_10mer_CG341.fq

cutadapt -m 50 -g ^CTCATCGGCG -o ./trimmed_barcode/CG342.fq sample_10mer_CG342.fq

cutadapt -m 50 -g ^TCTTAGCCCG -o ./trimmed_barcode/CG343.fq sample_10mer_CG343.fq

cutadapt -m 50 -g ^CGTGGAAGCG -o ./trimmed_barcode/CG344.fq sample_10mer_CG344.fq

cutadapt -m 50 -g ^GAGCTAGCCG -o ./trimmed_barcode/CG345.fq sample_10mer_CG345.fq

cutadapt -m 50 -g ^CCACCGATTA -o ./trimmed_barcode/TA114.fq sample_10mer_TA114.fq

cutadapt -m 50 -g ^CTTGGATATA -o ./trimmed_barcode/TA115.fq sample_10mer_TA115.fq

cutadapt -m 50 -g ^TGGTCTAATA -o ./trimmed_barcode/TA116.fq sample_10mer_TA116.fq

cutadapt -m 50 -g ^GAATTCTTTA -o ./trimmed_barcode/TA117.fq sample_10mer_TA117.fq

cutadapt -m 50 -g ^ACCAAGATTA -o ./trimmed_barcode/TA118.fq sample_10mer_TA118.fq

cutadapt -m 50 -g ^TTGCTATATA -o ./trimmed_barcode/TA119.fq sample_10mer_TA119.fq

cutadapt -m 50 -g ^GGTCGTAATA -o ./trimmed_barcode/TA120.fq sample_10mer_TA120.fq

cutadapt -m 50 -g ^AACGACATTA -o ./trimmed_barcode/TA121.fq sample_10mer_TA121.fq

cutadapt -m 50 -g ^CCAACGTATA -o ./trimmed_barcode/TA122.fq sample_10mer_TA122.fq

cutadapt -m 50 -g ^CTGTTAATTA -o ./trimmed_barcode/TA123.fq sample_10mer_TA123.fq

cutadapt -m 50 -g ^TGTGGCTATA -o ./trimmed_barcode/TA124.fq sample_10mer_TA124.fq

cutadapt -m 50 -g ^GAACCTATTA -o ./trimmed_barcode/TA125.fq sample_10mer_TA125.fq

cutadapt -m 50 -g ^ACCTAGTATA -o ./trimmed_barcode/TA126.fq sample_10mer_TA126.fq

cutadapt -m 50 -g ^CGTAGATTTA -o ./trimmed_barcode/TA127.fq sample_10mer_TA127.fq

cutadapt -m 50 -g ^TTCGTTAATA -o ./trimmed_barcode/TA128.fq sample_10mer_TA128.fq

cutadapt -m 50 -g ^GAGAACGTTA -o ./trimmed_barcode/TA129.fq sample_10mer_TA129.fq

cutadapt -m 50 -g ^ACACTGTATA -o ./trimmed_barcode/TA130.fq sample_10mer_TA130.fq

cutadapt -m 50 -g ^TTGGCTATTA -o ./trimmed_barcode/TA131.fq sample_10mer_TA131.fq

cutadapt -m 50 -g ^GGCTGAATTA -o ./trimmed_barcode/TA132.fq sample_10mer_TA132.fq

cutadapt -m 50 -g ^AATAACGATA -o ./trimmed_barcode/TA133.fq sample_10mer_TA133.fq

cutadapt -m 50 -g ^CCACGATATA -o ./trimmed_barcode/TA134.fq sample_10mer_TA134.fq

cutadapt -m 50 -g ^TGTTCGATTA -o ./trimmed_barcode/TA135.fq sample_10mer_TA135.fq

cutadapt -m 50 -g ^GTGGCTGATA -o ./trimmed_barcode/TA136.fq sample_10mer_TA136.fq

cutadapt -m 50 -g ^AACATGTTTA -o ./trimmed_barcode/TA137.fq sample_10mer_TA137.fq

cutadapt -m 50 -g ^CCACAAGTTA -o ./trimmed_barcode/TA138.fq sample_10mer_TA138.fq

cutadapt -m 50 -g ^TTGTTCAATA -o ./trimmed_barcode/TA139.fq sample_10mer_TA139.fq

cutadapt -m 50 -g ^CGAGGTTATA -o ./trimmed_barcode/TA140.fq sample_10mer_TA140.fq

cutadapt -m 50 -g ^GATACAATTA -o ./trimmed_barcode/TA141.fq sample_10mer_TA141.fq

cutadapt -m 50 -g ^ACCTACGTTA -o ./trimmed_barcode/TA142.fq sample_10mer_TA142.fq

cutadapt -m 50 -g ^GTTCTGTATA -o ./trimmed_barcode/TA143.fq sample_10mer_TA143.fq

cutadapt -m 50 -g ^TGGCGTGATA -o ./trimmed_barcode/TA144.fq sample_10mer_TA144.fq

cutadapt -m 50 -g ^AACGCATTTA -o ./trimmed_barcode/TA145.fq sample_10mer_TA145.fq

cutadapt -m 50 -g ^CCAAGCATTA -o ./trimmed_barcode/TA146.fq sample_10mer_TA146.fq

cutadapt -m 50 -g ^CTTGTGAATA -o ./trimmed_barcode/TA147.fq sample_10mer_TA147.fq

cutadapt -m 50 -g ^AGGCATTATA -o ./trimmed_barcode/TA148.fq sample_10mer_TA148.fq

cutadapt -m 50 -g ^TAATACGTTA -o ./trimmed_barcode/TA149.fq sample_10mer_TA149.fq

cutadapt -m 50 -g ^GCCACATTTA -o ./trimmed_barcode/TA150.fq sample_10mer_TA150.fq

cutadapt -m 50 -g ^CTGTTGGATA -o ./trimmed_barcode/TA151.fq sample_10mer_TA151.fq

cutadapt -m 50 -g ^GGTGCTATTA -o ./trimmed_barcode/TA152.fq sample_10mer_TA152.fq

cutadapt -m 50 -g ^TAACGATAACG -o ./trimmed_barcode/CG153.fq sample_11mer_CG153.fq

cutadapt -m 50 -g ^ACCAACGATCG -o ./trimmed_barcode/CG154.fq sample_11mer_CG154.fq

cutadapt -m 50 -g ^CTTGTGATACG -o ./trimmed_barcode/CG155.fq sample_11mer_CG155.fq

cutadapt -m 50 -g ^TCGTGTTATCG -o ./trimmed_barcode/CG156.fq sample_11mer_CG156.fq

cutadapt -m 50 -g ^GGAACAGTACG -o ./trimmed_barcode/CG157.fq sample_11mer_CG157.fq

cutadapt -m 50 -g ^AACCGGAATCG -o ./trimmed_barcode/CG158.fq sample_11mer_CG158.fq

cutadapt -m 50 -g ^TTGTACATTCG -o ./trimmed_barcode/CG159.fq sample_11mer_CG159.fq

cutadapt -m 50 -g ^CGTGTTGTACG -o ./trimmed_barcode/CG160.fq sample_11mer_CG160.fq

cutadapt -m 50 -g ^GAACACTAACG -o ./trimmed_barcode/CG161.fq sample_11mer_CG161.fq

cutadapt -m 50 -g ^ACCACAGTTCG -o ./trimmed_barcode/CG162.fq sample_11mer_CG162.fq

cutadapt -m 50 -g ^CTTGGTTAACG -o ./trimmed_barcode/CG163.fq sample_11mer_CG163.fq

cutadapt -m 50 -g ^TGGCTGATTCG -o ./trimmed_barcode/CG164.fq sample_11mer_CG164.fq

cutadapt -m 50 -g ^GAATCATAACG -o ./trimmed_barcode/CG165.fq sample_11mer_CG165.fq

cutadapt -m 50 -g ^ACCTAGGTTCG -o ./trimmed_barcode/CG166.fq sample_11mer_CG166.fq

cutadapt -m 50 -g ^TTGGTCAATCG -o ./trimmed_barcode/CG167.fq sample_11mer_CG167.fq

cutadapt -m 50 -g ^CGTAGTGAACG -o ./trimmed_barcode/CG168.fq sample_11mer_CG168.fq

cutadapt -m 50 -g ^GAACACATTCG -o ./trimmed_barcode/CG169.fq sample_11mer_CG169.fq

cutadapt -m 50 -g ^ACCACGTAACG -o ./trimmed_barcode/CG170.fq sample_11mer_CG170.fq

cutadapt -m 50 -g ^CTTGGAATTCG -o ./trimmed_barcode/CG171.fq sample_11mer_CG171.fq

cutadapt -m 50 -g ^TGGTCTTAACG -o ./trimmed_barcode/CG172.fq sample_11mer_CG172.fq

cutadapt -m 50 -g ^GAATTCGTACG -o ./trimmed_barcode/CG173.fq sample_11mer_CG173.fq

cutadapt -m 50 -g ^ACCGAATATCG -o ./trimmed_barcode/CG174.fq sample_11mer_CG174.fq

cutadapt -m 50 -g ^TGTCGGATACG -o ./trimmed_barcode/CG175.fq sample_11mer_CG175.fq

cutadapt -m 50 -g ^GTGACTGATCG -o ./trimmed_barcode/CG176.fq sample_11mer_CG176.fq

cutadapt -m 50 -g ^AACATCGTACG -o ./trimmed_barcode/CG177.fq sample_11mer_CG177.fq

cutadapt -m 50 -g ^CCACGTAATCG -o ./trimmed_barcode/CG178.fq sample_11mer_CG178.fq

cutadapt -m 50 -g ^CTGTAGTTACG -o ./trimmed_barcode/CG179.fq sample_11mer_CG179.fq

cutadapt -m 50 -g ^TCTGTAGTTCG -o ./trimmed_barcode/CG180.fq sample_11mer_CG180.fq

cutadapt -m 50 -g ^GGAACCTAACG -o ./trimmed_barcode/CG181.fq sample_11mer_CG181.fq

cutadapt -m 50 -g ^AACGATAATCG -o ./trimmed_barcode/CG182.fq sample_11mer_CG182.fq

cutadapt -m 50 -g ^TTGCTAATACG -o ./trimmed_barcode/CG183.fq sample_11mer_CG183.fq

cutadapt -m 50 -g ^GGTTGCTATCG -o ./trimmed_barcode/CG184.fq sample_11mer_CG184.fq

cutadapt -m 50 -g ^AACGTGGTTCG -o ./trimmed_barcode/CG185.fq sample_11mer_CG185.fq

cutadapt -m 50 -g ^CCAACGATACG -o ./trimmed_barcode/CG186.fq sample_11mer_CG186.fq

cutadapt -m 50 -g ^TTGCAAGAACG -o ./trimmed_barcode/CG187.fq sample_11mer_CG187.fq

cutadapt -m 50 -g ^AGCTGTTATCG -o ./trimmed_barcode/CG188.fq sample_11mer_CG188.fq

cutadapt -m 50 -g ^GATAACCTACG -o ./trimmed_barcode/CG189.fq sample_11mer_CG189.fq

cutadapt -m 50 -g ^CCACCGGATCG -o ./trimmed_barcode/CG190.fq sample_11mer_CG190.fq

cutadapt -m 50 -g ^CTGTGAATACG -o ./trimmed_barcode/CG191.fq sample_11mer_CG191.fq

cutadapt -m 50 -g ^AGTGCTTGTCG -o ./trimmed_barcode/CG192.fq sample_11mer_CG192.fq

cutadapt -m 50 -g ^CCTTGAAGGCG -o ./trimmed_barcode/CG247.fq sample_11mer_CG247.fq

cutadapt -m 50 -g ^ACAAGTAGCCG -o ./trimmed_barcode/CG346.fq sample_11mer_CG346.fq

cutadapt -m 50 -g ^TTCTACCGGCG -o ./trimmed_barcode/CG347.fq sample_11mer_CG347.fq

cutadapt -m 50 -g ^ATGGCGGCCCG -o ./trimmed_barcode/CG348.fq sample_11mer_CG348.fq

cutadapt -m 50 -g ^GGAACTAGGCG -o ./trimmed_barcode/CG349.fq sample_11mer_CG349.fq

cutadapt -m 50 -g ^CACCAATCGCG -o ./trimmed_barcode/CG350.fq sample_11mer_CG350.fq

cutadapt -m 50 -g ^ACTTGCCGCCG -o ./trimmed_barcode/CG351.fq sample_11mer_CG351.fq

cutadapt -m 50 -g ^TAGGTGGCCCG -o ./trimmed_barcode/CG352.fq sample_11mer_CG352.fq

cutadapt -m 50 -g ^GGAATACCGCG -o ./trimmed_barcode/CG353.fq sample_11mer_CG353.fq

cutadapt -m 50 -g ^CCTCACAGCCG -o ./trimmed_barcode/CG354.fq sample_11mer_CG354.fq

cutadapt -m 50 -g ^ATCTCTTCGCG -o ./trimmed_barcode/CG355.fq sample_11mer_CG355.fq

cutadapt -m 50 -g ^TGAGGTGGCCG -o ./trimmed_barcode/CG356.fq sample_11mer_CG356.fq

cutadapt -m 50 -g ^GAGACGTCGCG -o ./trimmed_barcode/CG357.fq sample_11mer_CG357.fq

cutadapt -m 50 -g ^CCTCTACGCCG -o ./trimmed_barcode/CG358.fq sample_11mer_CG358.fq

cutadapt -m 50 -g ^TTCTGCACGCG -o ./trimmed_barcode/CG359.fq sample_11mer_CG359.fq

cutadapt -m 50 -g ^GAGGATGGCCG -o ./trimmed_barcode/CG360.fq sample_11mer_CG360.fq

cutadapt -m 50 -g ^AGAATGACGCG -o ./trimmed_barcode/CG361.fq sample_11mer_CG361.fq

cutadapt -m 50 -g ^CCTCAATGGCG -o ./trimmed_barcode/CG362.fq sample_11mer_CG362.fq

cutadapt -m 50 -g ^ATCTCCGCCCG -o ./trimmed_barcode/CG363.fq sample_11mer_CG363.fq

cutadapt -m 50 -g ^TAGCGGCGCCG -o ./trimmed_barcode/CG364.fq sample_11mer_CG364.fq

cutadapt -m 50 -g ^GGAGAATCGCG -o ./trimmed_barcode/CG365.fq sample_11mer_CG365.fq

cutadapt -m 50 -g ^CCTTGTAGCCG -o ./trimmed_barcode/CG366.fq sample_11mer_CG366.fq

cutadapt -m 50 -g ^TTCACGGCGCG -o ./trimmed_barcode/CG367.fq sample_11mer_CG367.fq

cutadapt -m 50 -g ^GAGGTCCGCCG -o ./trimmed_barcode/CG368.fq sample_11mer_CG368.fq

cutadapt -m 50 -g ^AGAACTCCGCG -o ./trimmed_barcode/CG369.fq sample_11mer_CG369.fq

cutadapt -m 50 -g ^CCTCTGAGGCG -o ./trimmed_barcode/CG370.fq sample_11mer_CG370.fq

cutadapt -m 50 -g ^TTCTAATGCCG -o ./trimmed_barcode/CG371.fq sample_11mer_CG371.fq

cutadapt -m 50 -g ^AGTGGCGCGCG -o ./trimmed_barcode/CG372.fq sample_11mer_CG372.fq

cutadapt -m 50 -g ^GAGAACTCCCG -o ./trimmed_barcode/CG373.fq sample_11mer_CG373.fq

cutadapt -m 50 -g ^CCACTTAGCCG -o ./trimmed_barcode/CG374.fq sample_11mer_CG374.fq

cutadapt -m 50 -g ^TTCTCACCGCG -o ./trimmed_barcode/CG375.fq sample_11mer_CG375.fq

cutadapt -m 50 -g ^AGGTGTGGCCG -o ./trimmed_barcode/CG376.fq sample_11mer_CG376.fq

cutadapt -m 50 -g ^GAAGCGTGGCG -o ./trimmed_barcode/CG377.fq sample_11mer_CG377.fq

cutadapt -m 50 -g ^CCTCGCACGCG -o ./trimmed_barcode/CG378.fq sample_11mer_CG378.fq

cutadapt -m 50 -g ^TTCATACGCCG -o ./trimmed_barcode/CG379.fq sample_11mer_CG379.fq

cutadapt -m 50 -g ^GCGGATGCGCG -o ./trimmed_barcode/CG380.fq sample_11mer_CG380.fq

cutadapt -m 50 -g ^AGAATGCGCCG -o ./trimmed_barcode/CG381.fq sample_11mer_CG381.fq

cutadapt -m 50 -g ^CACCGCTCCCG -o ./trimmed_barcode/CG382.fq sample_11mer_CG382.fq

cutadapt -m 50 -g ^TTGTAAGCGCG -o ./trimmed_barcode/CG383.fq sample_11mer_CG383.fq

cutadapt -m 50 -g ^GGTGCGAGCCG -o ./trimmed_barcode/CG384.fq sample_11mer_CG384.fq

cutadapt -m 50 -g ^TAACGATAATA -o ./trimmed_barcode/TA153.fq sample_11mer_TA153.fq

cutadapt -m 50 -g ^ACCAACGATTA -o ./trimmed_barcode/TA154.fq sample_11mer_TA154.fq

cutadapt -m 50 -g ^CTTGTGATATA -o ./trimmed_barcode/TA155.fq sample_11mer_TA155.fq

cutadapt -m 50 -g ^TCGTGTTATTA -o ./trimmed_barcode/TA156.fq sample_11mer_TA156.fq

cutadapt -m 50 -g ^GGAACAGTATA -o ./trimmed_barcode/TA157.fq sample_11mer_TA157.fq

cutadapt -m 50 -g ^AACCGGAATTA -o ./trimmed_barcode/TA158.fq sample_11mer_TA158.fq

cutadapt -m 50 -g ^TTGTACATTTA -o ./trimmed_barcode/TA159.fq sample_11mer_TA159.fq

cutadapt -m 50 -g ^CGTGTTGTATA -o ./trimmed_barcode/TA160.fq sample_11mer_TA160.fq

cutadapt -m 50 -g ^GAACACTAATA -o ./trimmed_barcode/TA161.fq sample_11mer_TA161.fq

cutadapt -m 50 -g ^ACCACAGTTTA -o ./trimmed_barcode/TA162.fq sample_11mer_TA162.fq

cutadapt -m 50 -g ^CTTGGTTAATA -o ./trimmed_barcode/TA163.fq sample_11mer_TA163.fq

cutadapt -m 50 -g ^TGGCTGATTTA -o ./trimmed_barcode/TA164.fq sample_11mer_TA164.fq

cutadapt -m 50 -g ^GAATCATAATA -o ./trimmed_barcode/TA165.fq sample_11mer_TA165.fq

cutadapt -m 50 -g ^ACCTAGGTTTA -o ./trimmed_barcode/TA166.fq sample_11mer_TA166.fq

cutadapt -m 50 -g ^TTGGTCAATTA -o ./trimmed_barcode/TA167.fq sample_11mer_TA167.fq

cutadapt -m 50 -g ^CGTAGTGAATA -o ./trimmed_barcode/TA168.fq sample_11mer_TA168.fq

cutadapt -m 50 -g ^GAACACATTTA -o ./trimmed_barcode/TA169.fq sample_11mer_TA169.fq

cutadapt -m 50 -g ^ACCACGTAATA -o ./trimmed_barcode/TA170.fq sample_11mer_TA170.fq

cutadapt -m 50 -g ^CTTGGAATTTA -o ./trimmed_barcode/TA171.fq sample_11mer_TA171.fq

cutadapt -m 50 -g ^TGGTCTTAATA -o ./trimmed_barcode/TA172.fq sample_11mer_TA172.fq

cutadapt -m 50 -g ^GAATTCGTATA -o ./trimmed_barcode/TA173.fq sample_11mer_TA173.fq

cutadapt -m 50 -g ^ACCGAATATTA -o ./trimmed_barcode/TA174.fq sample_11mer_TA174.fq

cutadapt -m 50 -g ^TGTCGGATATA -o ./trimmed_barcode/TA175.fq sample_11mer_TA175.fq

cutadapt -m 50 -g ^GTGACTGATTA -o ./trimmed_barcode/TA176.fq sample_11mer_TA176.fq

cutadapt -m 50 -g ^AACATCGTATA -o ./trimmed_barcode/TA177.fq sample_11mer_TA177.fq

cutadapt -m 50 -g ^CCACGTAATTA -o ./trimmed_barcode/TA178.fq sample_11mer_TA178.fq

cutadapt -m 50 -g ^CTGTAGTTATA -o ./trimmed_barcode/TA179.fq sample_11mer_TA179.fq

cutadapt -m 50 -g ^TCTGTAGTTTA -o ./trimmed_barcode/TA180.fq sample_11mer_TA180.fq

cutadapt -m 50 -g ^GGAACCTAATA -o ./trimmed_barcode/TA181.fq sample_11mer_TA181.fq

cutadapt -m 50 -g ^AACGATAATTA -o ./trimmed_barcode/TA182.fq sample_11mer_TA182.fq

cutadapt -m 50 -g ^TTGCTAATATA -o ./trimmed_barcode/TA183.fq sample_11mer_TA183.fq

cutadapt -m 50 -g ^GGTTGCTATTA -o ./trimmed_barcode/TA184.fq sample_11mer_TA184.fq

cutadapt -m 50 -g ^AACGTGGTTTA -o ./trimmed_barcode/TA185.fq sample_11mer_TA185.fq

cutadapt -m 50 -g ^CCAACGATATA -o ./trimmed_barcode/TA186.fq sample_11mer_TA186.fq

cutadapt -m 50 -g ^TTGCAAGAATA -o ./trimmed_barcode/TA187.fq sample_11mer_TA187.fq

cutadapt -m 50 -g ^AGCTGTTATTA -o ./trimmed_barcode/TA188.fq sample_11mer_TA188.fq

cutadapt -m 50 -g ^GATAACCTATA -o ./trimmed_barcode/TA189.fq sample_11mer_TA189.fq

cutadapt -m 50 -g ^CCACCGGATTA -o ./trimmed_barcode/TA190.fq sample_11mer_TA190.fq

cutadapt -m 50 -g ^CTGTGAATATA -o ./trimmed_barcode/TA191.fq sample_11mer_TA191.fq

cutadapt -m 50 -g ^AGTGCTTGTTA -o ./trimmed_barcode/TA192.fq sample_11mer_TA192.fq

cutadapt -m 50 -g ^CCTTGAAGGTA -o ./trimmed_barcode/TA247.fq sample_11mer_TA247.fq
